# Molecular detection of *mecA* gene from methicillin-resistant *Staphylococcus aureus* isolated from clinical and environmental samples and its potential inhibition by phytochemicals using in vitro and in silico approach

**DOI:** 10.1007/s40203-024-00297-y

**Published:** 2025-02-10

**Authors:** Mohammed M. Mohammed, Mustafa Alhaji Isa, Mustapha B. Abubakar, Abubakar Sadiq Baba Dikwa, Abidemi Paul Kappo

**Affiliations:** 1 Department of Microbiology, Faculty of Life Sciences, University of Maiduguri, Maiduguri, Nigeria; 2 Molecular Biophysics and Structural Biology (MBSB) Group, Department of Biochemistry, University of Johannesburg, Auckland Park Kingsway Campus, Johannesburg, 2006 South Africa; 3 Department of Veterinary Microbiology, Faculty of Veterinary Medicine, University of Maiduguri, Maiduguri, Nigeria; 4 Department of Medical Microbiology, University of Maiduguri Teaching Hospital, Maiduguri, Nigeria

**Keywords:** *Acacia nilotica*, *Mangifera indica*, MRSA, Phytochemical, MIC, Docking, MD simulation

## Abstract

The increasing prevalence of Methicillin-resistant *Staphylococcus aureus* (MRSA) has posed significant challenges in clinical and environmental settings. MRSA's resistance is attributed to the *mecA* gene, which encodes the penicillin-binding protein 2a (PBP2a), conferring resistance to β-lactam antibiotics. This study aimed to molecularly detect the *mecA* gene in MRSA isolates from clinical and environmental samples and identify potential inhibitors of PBP2a using in vitro and in silico approaches. A total of 180 samples were collected, isolating 64 *Staphylococcus aureus* strains, of which 10 (37%) were confirmed as MRSA. Molecular detection confirmed the presence of the *mecA* gene in these isolates. Phytochemical analysis of *Acacia nilotica* and *Mangifera indica* extracts revealed bioactive compounds with antimicrobial properties. In vitro antimicrobial testing showed the plant extracts demonstrated significant inhibitory effects against MRSA, with minimum inhibitory concentrations (MIC) ranging from 0 to 6.20 mg/mL. Molecular docking and molecular dynamics simulation analyses identified three compounds (CID_628694, CID_546930, and CID_620007) with strong binding affinities and stable interactions with PBP2a, suggesting their potential as novel inhibitors. These findings highlight the therapeutic potential of phytochemicals in combating MRSA and provide a basis for future experimental validations.

## Introduction

The escalating incidence of infections caused by antibiotic-resistant bacteria presents a formidable global health challenge. Among the most concerning pathogens is *Staphylococcus aureus*, a Gram-positive bacterium that has evolved remarkable adaptability to various antibacterial agents through genetic mutation, horizontal gene transfer, and selection pressure. This adaptability has enabled *S. aureus* to cause a broad spectrum of diseases, ranging from relatively minor skin infections to severe conditions like pneumonia, osteomyelitis, endocarditis, and sepsis (Chambers 2001). A critical concern is the rise of methicillin-resistant *S. aureus* (MRSA), a strain that resists nearly all β-lactam antibiotics, making it particularly difficult to treat. MRSA has emerged as one of the most virulent human pathogens, significantly contributing to hospital-acquired infections (HAIs), leading to increased morbidity, mortality, and healthcare costs (Kiran et al. 2008). MRSA is not limited to hospital environments; community-associated MRSA (CA-MRSA) infections have also become prevalent, affecting individuals with no recent hospital exposure (Kobayashi et al. 2014). This expansion into community settings emphasizes the urgent need for novel therapeutic strategies to manage MRSA in healthcare and public domains. The emergence of vancomycin-resistant *S. aureus* (VRSA), which further limits treatment options, exacerbates this issue (Kennedy et al. 2008). Since its first clinical identification in the 1960s, MRSA has continued to evolve, spreading rapidly within hospitals, communities, and even livestock. The most prominent strains, such as clonal complex 30 (CC30) in Europe and USA300 in North America, have been responsible for several MRSA infection waves, with varying regional patterns of spread (Turner et al. 2019).

The molecular basis for MRSA's resilience primarily lies in acquiring the *mecA* gene housed within the staphylococcal cassette chromosome mec (SCCmec). This gene encodes penicillin-binding protein 2a (PBP2a), which has a low affinity for β-lactam antibiotics, rendering them ineffective in disrupting the bacterium's cell wall synthesis (Ferreira et al. 2011). The global distribution of mecA-positive MRSA strains emphasizes the adaptability of *S. aureus* in acquiring resistance mechanisms through independent evolutionary events. Furthermore, the production of various exotoxins, including toxic shock syndrome toxin (TSST-1), exfoliative toxins, and enterotoxins, contributes to the bacterium's pathogenicity, leading to severe clinical manifestations (Aarestrup et al. 2010). Despite decades of effort, MRSA remains a significant cause of mortality, with current antibiotics unable to address its growing resistance fully. The introduction of antibiotics in the mid-twentieth century reduced mortality from *S. aureus* bacteremia. However, resistance has eroded these gains, and mortality rates for MRSA-related infections remain between 15 and 50%, depending on the setting and patient population (Turner et al. 2019). This has prompted a need for continuous surveillance of resistance genes, such as *mecA*, and the exploration of alternative therapeutic agents.

Although vancomycin was once considered a gold standard for treating MRSA infections, increasing resistance to vancomycin and other last-line antibiotics like fluoroquinolones has left clinicians with limited options (Tiwari et al. 2009). Fluoroquinolone resistance in *S. aureus* (FRSA), driven by mutations in target genes and the production of efflux pumps, has further compounded the therapeutic challenge, highlighting the urgency of developing new antimicrobials (Gade and Qazi 2013). Given the limitations of current antibiotics, there is growing interest in discovering novel therapeutic agents that can bypass traditional resistance mechanisms. One promising avenue is the investigation of naturally occurring compounds with antimicrobial properties. Natural products have historically served as the foundation for many antimicrobial drugs, offering diverse chemical scaffolds that can be modified for enhanced efficacy and reduced toxicity. Phytochemicals have shown potential as alternative or complementary treatments to conventional antibiotics, particularly in the fight against drug-resistant bacteria like MRSA. The unique chemical structures of these compounds often allow them to interact with bacterial targets in ways that differ from synthetic antibiotics, thereby reducing the likelihood of resistance development.

Natural products have garnered significant attention in antimicrobial research due to their unique advantages over synthetic chemical compounds (ME et al. 2024). Unlike many synthetic agents, natural products often exhibit diverse and complex chemical structures that can target pathogens through novel mechanisms, reducing the likelihood of resistance development (Kalaba et al. 2024). These compounds are biosynthesized by living organisms, reflecting millions of years of evolutionary optimization for biological activity, which can offer higher specificity and potency against bacterial targets. Moreover, natural products frequently possess multiple pharmacophores, enabling them to interact with diverse molecular targets simultaneously, a less common feature in chemically synthesized drugs (Elbestawy et al. 2023).

To address the escalating crisis of MRSA and other drug-resistant pathogens, research is increasingly focused on identifying compounds that can inhibit the function of vital bacterial proteins, such as PBP2a. Inhibitors that can disrupt the activity of PBP2a would effectively restore the efficacy of β-lactam antibiotics, providing a novel approach to overcoming MRSA resistance. Moreover, combining such inhibitors with existing antibiotics could lead to synergistic effects, enhancing treatment outcomes and reducing the emergence of further resistance. This study is positioned within the broader context of the ongoing search for novel antimicrobial agents capable of tackling antibiotic-resistant bacteria. By exploring naturally derived compounds, particularly those with the potential to inhibit resistance mechanisms like PBP2a, this research aims to contribute to the development of “next-generation therapies” for MRSA and other resistant pathogens. The investigation into natural compounds and their mechanisms of action represents a novel and timely approach to address one of the most pressing challenges in infectious disease management. Therefore, this study's novelty lies in its focus on unravelling the potential of naturally derived inhibitors to combat MRSA, addressing the urgent need for alternative treatments in the face of growing global antibiotic resistance.

## Materials and methods

### Collection of samples and phenotypic evaluation

The study was conducted at the University of Maiduguri Teaching Hospital (UMTH) between March 2021 and October 2021. Ethical clearance and approval were obtained from the Research and Ethics Committee of the University of Maiduguri Teaching Hospital (UMTH/REC/19/345). A total of 180 samples were procured from clinical specimens (nasal swabs) and environmental samples (collected from the hospital environment, hospital beds, and wastewater). Swab sticks and 200ml sterile bottles were appropriately labelled with unique participant study numbers. These collected samples were promptly transported to the laboratory and streaked onto Mannitol Salt agar plates (Hi-Media Laboratories, Mumbai). The plates were then incubated at 37 °C for 24–48 h, and Gram smears were prepared from distinct types of colonies. The identification of *Staphylococcus aureus* colonies was based on Gram reactions, colony characteristics, pigment production, catalase and coagulase tests, and DNase testing (Dauner et al. 2010).

### Assessment of antimicrobial susceptibility

The antimicrobial resistance profile of the *S. aureus* isolates was assessed using a modified Kirby Bauer disc diffusion method with the following antibiotics: Ampiclox (30 ug), Amoxicillin (30 ug), Cefuroxime (20 ug), Ceftriaxone (25 ug), Erythromycin (10 ug), Cotrimoxazole (30 ug), Ciprofloxacin (10 ug), Pefloxacin (10 ug), Streptomycin (30 ug), and Gentamycin (10 ug). The antibiotics used in this study were sourced from Sigma-Aldrich, a reputable supplier of pharmaceutical-grade chemicals and microbiological reagents. In this procedure, a circular piece of filter paper infused with antibiotics was positioned onto a Mueller Hinton agar plate that had been uniformly inoculated with the *Staphylococcus aureus* test strain. The antibiotic then diffuses from the paper disc into the agar medium, inhibiting bacterial growth at a certain distance from the disc. This distance is measured as the Zone of Inhibition in millimeters, and the resistance pattern is determined based on established standard methods (CLSI 2017).

### Detection of methicillin-resistant *S. aureus*

The procedure adhered to the recommendations of Clinical and Laboratory Standard Institute (CLSI 2017). Pure cultures were established by culturing the control strain and individual *S. aureus* isolates separately. These cultures were inoculated into nutrient broth and incubated for 24 h at 37 °C. Following incubation, the cultures were diluted with 0.85% saline solution, and the turbidity was adjusted to match the 0.5 McFarland standard for uniformity in bacterial density, ensuring accurate comparison and subsequent testing. Inoculations onto Mueller-Hinton agar supplemented with 2% NaCl (Oxoid Ltd.) for antimicrobial susceptibility testing. The bacterial inoculum was evenly spread over the agar surface using a sterile cotton swab. Antibiotic susceptibility was assessed using antibiotic discs placed on the agar plate. Specifically, Oxacillin discs (1 μg), Methicillin discs (5 μg), and Cefoxitin discs (30 μg), all sourced from Oxoid Ltd., were carefully positioned onto the inoculated agar plate using sterile forceps. The plates were then incubated aerobically at 35 °C for 24 h. The inhibition zones were measured after incubation to determine the susceptibility pattern of the bacterial isolates. The diameter of the inhibitory zone for each isolate was measured and compared to the standard. The standardized antibiotic susceptibility testing of the reference strain was done using *S. aureus* MTCC 87/ATCC25923. All isolates resistant to Cefoxitin were later confirmed as MRSA using Oxoid Brilliant Green Agar.

### DNA extraction and quantification

In adherence to the manufacturer's guidelines, bacterial DNA was isolated using the AccuPrep® Genomic DNA Extraction Kit (Bioneer Corporation, USA). A bacterial colony was initially transferred to a 1.5 mL tube containing 20 μL of cell lysis buffer. Subsequently, 20 μL of binding buffer was added, and the mixture was incubated at 60 °C for 10 min, followed by centrifugation to eliminate any residual droplets. After adding 10 μL of isopropanol, the lysate was moved to a binding column tube for centrifugation. The subsequent steps involved washings, and the final elution of the sample was carried out using 200 μL of elution buffer in a fresh tube. Finally, 50 μL of the supernatant was stored at -20 °C, and 5µL was used for each PCR assay.

Polymerase chain reaction (PCR) for *mecA* gene

The *mecA* gene was PCR-amplified in a 20μL reaction at the Nigerian Institute for Trypanosomiasis Research (NITR) in Kaduna. This amplification process utilized the AccuPower® HotStart PCR PreMix and employed prokaryotic primers designed for the *mecA* gene: *mecA-F* 5′-AAAATCGATGGTAAAGGTTGGC-3′

*mecA-R* 5′-AGTTCTGCAGTACCGGATTTGC-3′.

In this experiment, the Master-mix buffer consists of the following components: 5 μL of PCR buffer, 0.25 μL of polymerase, 1.5 μL of MgCl_2_, 2.5 μL of dNTPs, 3.0 μL of primer, 3 μg of DNA template, and ultrapure sterile water (9.75 μL), making a total volume of 25 μL. The PCR cycling conditions involve an initial denaturation cycle at 95 °C for 5 min, followed by 35 cycles of denaturation at 94 °C for 1 min, annealing at 46 °C for 45 seconds, and extension at 72 °C for 1 min, concluding with a final extension step lasting 10 min at 72 °C (Wu et al. 2012). Following amplification, the resulting gene products were examined by electrophoresis on a 1% agarose gel in 100 mL TBE buffer, with a DNA ladder of known 1000bp size. The gel was stained with a 0.5 μg/mL ethidium bromide solution for 15 min and visualized using a gel imaging system (transilluminator). Samples displaying a single distinct band of the expected length on the gel were used to determine their respective base pair sizes.

### Collection and extraction of plant material

Fully mature leaves and fruits of *Acacia nilotica* and *Mangifera indica*, harvested from the University of Maiduguri botanical garden in Nigeria, were identified and authenticated by a taxonomist at the Department of Biological Sciences' Herbarium Unit. Voucher specimens (reference numbers MCB/21/031, MCB/21/032, and MCB/21/033) were stored in the University of Maiduguri's Department of Microbiology Research Laboratory. These plants were selected for their documented traditional medicinal uses and known bioactive compounds, which have demonstrated antimicrobial and therapeutic properties in previous studies. Their availability and relevance to the local context further supported their inclusion in this research.The collected plant materials were disease-free and devoid of unwanted parts. After washing to remove dust, they were shade-dried for three days and then ground into fine powder using a sterile mortar and pestle. This powdered sample was subjected to sequential extraction with chloroform, alcohol, and distillation via hot continuous percolation in a Soxhlet apparatus for 24 h. The resulting extract was concentrated using a rotary evaporator and freeze-dried in a lyophilizer to obtain dry powder (Harborne 1984). This dried powder was subjected to both preliminary phytochemical analysis and Gas chromatography-Mass Spectrometry (GC–MS) analysis.

### Qualitative phytochemical screening

Phytochemical screening was conducted using standard procedures to detect various bioactive compounds in the crude extract and partitioned fractions of *Acacia nilotica* and *Mangifera indica* plants. The screening was designed to identify the presence of critical phytochemicals, including alkaloids, flavonoids, glycosides, tannins, phlorotannins, terpenoids, and carbohydrates. The methodology followed established protocols as described by Brain et al. (1975), Vishnoi (1979), Sofowora (1993).

For alkaloid detection, a few drops of Mayer's reagent were added to a small volume of the extract, and the formation of a yellow or cream-coloured precipitate confirmed the presence of alkaloids. Flavonoids were detected using alkaline reagent tests, and yellow indicated their presence. Glycosides were tested by adding Fehling's solution, with a red precipitate confirming their presence. Tannins were screened using 1% lead acetate solution, which produced a precipitate in the presence of tannins. Phlobatannins were identified by heating the extracts with 2% hydrochloric acid, where a red precipitate indicated their presence. Terpenoids were screened using the Liebermann-Burchard test, where the appearance of a green or blue colour indicated their presence. Finally, the presence of carbohydrates was confirmed by performing a Molisch test, where a purple ring formed upon adding α-naphthol and concentrated sulfuric acid.

## Assessment of antimicrobial activity

The antibacterial efficacy of *A. nilotica* and *M. indica* leaves extracts were assessed using the agar-well diffusion technique (Okeke et al. 2001). Mueller-Hinton agar medium was thoroughly mixed with 100 µL of a standardized microbial stock suspension (1 × 10^8^) and poured into sterile Petri plates. Three wells, each 8 mm in diameter, were aseptically created in every Petri plate using a sterile cork borer No. 4. Subsequently, 100 µL, 75 µL, 50 µL and 25 µL of the respective 250 µg per well extracts were introduced into two of these wells. In the third well, 100 µL of a vancomycin solution at a concentration of 5 µg was added as a control. The plates were then incubated at 37 °C overnight to facilitate bacterial growth. Following incubation, the diameter of the inhibitory zones was measured and recorded for each plant extract.

### Minimal inhibitory concentration (MIC) and minimal bactericidal concentration (MBC) of extracts from *A. nilotica* and *M. indica* leaves and fruits

The MIC was determined through the broth dilution method, where crude extracts were subjected to two-fold serial dilutions (ranging from 0 to 2000 µg/mL) in Mueller-Hinton broth, with appropriate antibiotics as controls. A 24-hour-old microbial suspension in Mueller-Hinton broth was adjusted to a turbidity matching 0.5 McFarland standards (equivalent to 2.4 × 10^8^ cfu/ml) using a spectrophotometer at 625 nm. For the broth dilution tests, 0.1 mL of a standardized bacterial suspension (10^8^ cfu/mL) was added to tubes containing varying concentrations (ranging from 0 to 2000 µg/mL) and then incubated at 37 °C. The MIC was determined to be the lowest concentration, with no observable macroscopic growth. Dilutions from this test were streaked onto Mueller-Hinton agar plates, beginning with the tube that showed no growth. The plates were subsequently incubated at 37 °C for overnight growth. The minimum bactericidal concentration (MBC) was determined as the lowest extract concentration, where no discernible growth occurred on the agar plates after 24 h. All experiments were conducted in triplicate.

### Quantitative phytochemical analysis using GC–MS

The quantitative analysis of the active phytochemical compounds was carried out using GC–MS to provide precise identification and concentration of bioactive compounds in the *A. nilotica* and *M. indica* extracts. The GC–MS analysis was conducted using an Agilent Technologies 7890B GC system coupled with an Agilent 5977 MSD mass spectrometer (MS) for detailed compound identification. A 2 mL aliquot of the extract was injected into the gas chromatograph (GC) using a splitless injection mode to maximize compound detection sensitivity. The GC was equipped with a DB5-MS capillary column (30 m length, 0.25 mm internal diameter, and 0.25 μm film thicknesses) suitable for separating complex mixtures of volatile compounds. Helium, with a high purity of 99.999%, was used as the carrier gas at a constant flow rate of 1 mL/min. The injector temperature was maintained at 200 °C to ensure complete vapourization of the sample. The oven temperature program was optimized to separate the extract's phytochemicals efficiently. Initially, the temperature was held at 70 °C for 3 min, followed by a linear increase of 5 °C/min until reaching 380 °C, where it was held for 1 min. A cooling phase was then initiated to bring the oven back to 70 °C, followed by a 5-min delay to stabilize the system before the next run (Kadhim and AL-Shammaa, 2014).

Mass spectrometric detection was performed using electron ionization (EI) mode at 70 eV, a standard setting for efficient fragmentation of organic molecules. The mass spectrometer was set to scan over 50–500 m/z to capture a broad spectrum of molecular weights. The ion source and quadrupole temperatures were maintained at 230 °C and 150 °C respectively, to ensure optimal ionization and resolution of the compounds. The transfer line was set at 250 °C to prevent condensation of volatile compounds. The quantification of the active compounds was performed using ion trace integration, where the area under the curve (AUC) for each detected compound was calculated. The integration process was executed with the "Find Target" method of the MassLab software, enabling the precise identification of each compound based on its retention time and fragmentation pattern. Calibration curves were generated for known standards of each identified compound, allowing the calculation of compound concentrations within the sample by correlating the peak area with the standard curve. To ensure accuracy and reproducibility, each sample was injected in triplicate, and the average peak area was used for concentration determination.

The mass spectra of the detected compounds were compared against the National Institute of Standards and Technology (NIST) database, which contains reference spectra for a wide range of known compounds. The software matched the spectra of the unknown compounds to those stored in the NIST library, enabling the identification of the molecular weight, structure, and name of each phytochemical. Compounds with a match factor above 90% were considered reliable identifications. For reproducibility, a blank sample was run before each sample injection to ensure no carryover between runs. Additionally, calibration standards of known concentration were injected at the beginning and end of the sample sequence to account for potential instrument drift and verify the accuracy of the quantification process. All identified compounds and their corresponding concentrations were recorded for further analysis.

### Analysis of physicochemical and medicinal chemistry characteristics

The analysis of physiochemical properties and medicinal chemistry characteristics for all compounds identified through GC-MS was conducted and screened using DataWarrior tool Version 4.6 (Sander et al. 2015), ADMETLAB 2.0 (Xiong et al. 2021), and SwissADME (Daina et al. 2017). Physiochemical and medicinal chemistry parameters assessed included molecular weight, hydrogen bond donors, hydrogen bond acceptors, Water-Oil Partition Coefficient (WLogP), Molecular Logarithm of the Partition Coefficient (MLogP), molar refractivity, number of rotatable bonds, topological polar surface area (TPSA), and the identification of potentially problematic compounds (PAINS) (Isa et al. 2020; Isa et al. 2018).

### Pharmacokinetic analysis

Evaluating a compound's pharmacokinetic properties, encompassing absorption, distribution, metabolism, excretion, and toxicity, is crucial in drug development. Many substances with unfavourable pharmacokinetic profiles never progress to clinical trials. Therefore, assessing these attributes is imperative when searching for new compounds. In this study, we utilized the AdmetSAR tool (Cheng et al. 2012) and DataWarrior tool (Lipinski et al. 1997; Veber et al. 2002) to appraise the pharmacokinetic characteristics of phytochemical constituents with strong binding affinities. These characteristics encompassed cytochrome P450 (CYP450 2D6) inhibitory activity, blood–brain barrier (BBB) penetration, human intestinal absorption (HIA), aqueous solubility, plasma protein binding, as well as factors related to mutagenicity, tumorigenicity, irritation, and reproductive effects. This assessment selected phytochemical components that exhibited desirable pharmacokinetic characteristics as potential novel inhibitors of the *mecA* gene product.

### Preparation of the crystal structure of the product encoded by the *mecA* gene

The crystal structure of Penicillin Binding Protein 2a (*mecA* gene product) was obtained from the Protein Data Bank (PDB_ID:1VQQ). The protein's binding site was prepared by eliminating all non-amino acid components, and chain A was selected for docking studies. The structure underwent a refinement process that involved clearing, addressing incomplete residues by adding missing atoms, eliminating alternate conformations, determining bond orders, optimizing and stabilizing side chains, resolving improper chirality, validating disulfide bonds, rectifying steric clashes, and overall protein optimization. Hydrogen atoms were introduced to the enzyme, and its energy was computed and minimized using SPDBV Version 4.10 (Johansson et al. 2012).

### Computational molecular docking assessment

Molecular docking enables us to comprehend how a protein interacts with a ligand or another protein. In this study, the AutoDock 4.2 program, an extension of Python Molecular Viewer, was utilized to prepare the protein and ligand for docking. The compound with the most favourable physicochemical and pharmacokinetics properties was selected for the docking studies (Imran et al. 2018). The ligands were prepared by retrieving their 2D structures from the PubChem database and converting them into 3D conformations using OpenBabel. The 3D ligand structures were optimized through energy minimization using the MMFF94 force field to achieve a stable conformation. During preparation, torsional flexibility was introduced by defining rotatable bonds to allow the ligand to adopt multiple conformations during docking.

Docking investigations were conducted using the active molecules from the phytochemicals. Cl^−^ and Cd^2+^ ions (cofactors) were docked with PBP2A to compare their binding affinities with those of the chosen ligands. The protein was subjected to automated docking employing a Lamarckian genetic algorithm (Morris et al. 1998). While the ligands' side chains and torsion bonds were allowed to rotate, PBP2a remained rigid. Polar hydrogen atoms were added to PBP2a, and Gasteiger charges were computed (Gasteiger and Marsili, 1980). The docking procedure encompassed 10 runs, a maximum of 2,500,000 evaluations, and 27,000 generations. For PBP2a, non-amino acid residues and water molecules were removed, and a cubic docking box was employed with coordinates x = 3.031, y = 35.614, and z = 14.419. The grid had dimensions of 60 × 60 × 60 with a grid spacing of 0.375.

### Analysis of molecular dynamics simulation (MD)

Molecular dynamics simulations of PBP2a bound to ligands was conducted using the AMBERTOOLS22 MD simulation software (Case et al. 2023). Hydrogen atoms were explicitly added through a 3D protonation approach. Coordinate and topology files for the complexes were generated using the tleap tool, and any missing ligand attributes were added using Antechamber. Force fields, including ff12SB for the ligand and GAFF for the protein, were assigned using Tleap. The entire system was placed in an octahedral box with ten dimensions, surrounded by a buffer solution, and neutralized with sodium ions. A two-step minimization process was applied to eliminate structural artifacts, involving 5000 steps of conjugate gradient and 5000 steps of steepest descent with constraints set at 544 kcal/mol/ on the complexes. After constraint release, a reduction phase was conducted with 2500 steepest descent and 2500 conjugate gradient steps. The system was heated from an initial temperature of zero to a final temperature of 300 degrees through 100,000 steps using the Langevin dynamics temperature regulation method, with no pressure control. The MD simulation employed a time step of 2fs and constant temperature and pressure settings (300K and 1 Atm) with the Berendsen barostat for pressure regulation. The simulation extended for 50 ns, during which SHAKE was applied to restrict hydrogen bonding. The stability of the protein-ligand complex was assessed through root mean square deviation (RMSD), and the mobility of specific amino acids in relation to their native positions was monitored using root mean square fluctuation (RMSF). The CPPTRAJ component of the AMBERTOOLS22 package was employed for this analysis.

## Results and discussion

### Isolation and molecular detection of *mecA* gene

*S. aureus* is ubiquitous and, despite being a regular inhabitant of the human anterior nares, skin, and genital tract, it can cause infections in virtually any organ or system within the body. Infections such as wounds, skin issues, soft tissue infections, bloodstream infections, and lower urinary tract infections are particularly prevalent. Antibiotic resistance has emerged as a major microbiological issue in the twenty-first century. *S. aureus* has consistently posed difficulties for antimicrobial treatment, with the development of resistant strains often coinciding with the introduction of new antimicrobial medications. As a result, it is imperative to regularly assess the susceptibility of *S. aureus* to antibiotics to effectively address infections in both healthcare settings and in the community, while also identifying emerging resistance trends (Olowe et al. 2013).”In this study, *S. aureus* was isolated from various clinical and environmental samples collected. Out of the 180 samples cultured, 64 samples tested positive for *Staphylococcus aureus*, resulting in a prevalence of 35.5% in this study. Among the 64 *S. aureus* strains that were isolated, 21 (32.8%) originated from swab samples collected from in-patients and outpatients, 7 (10.9%) from hospital beds, and 15 (23.4%) from sewage waste. Among the positive *S. aureus* isolates (n = 64), 27 (42.1%) resisted the 10 antibiotics used for susceptibility testing. Of these, 7 isolates (25.9%) came from inpatients, 6 (22.2%) each from outpatients and hospital beds, and 8 (29.6%) from hospital wastewater (Table 1).

**Table 1:** Distribution of *S. aureus* isolates exhibiting antimicrobial resistance

S/no.	Source of isolates	Total positive isolates (n)	Resistant isolates (n)	Resistant isolates (%)
1	In-patients	21	7	33.3
2	Out-patients	21	6	28.5
3	Hospital equipment (Bed)	7	6	85.7
4	Hospital wastewater (Sewage)	15	8	53.3
5	Total	64	27	42.1

Among the 27 *S. aureus* isolates displaying resistance to multiple drugs, which were subsequently examined for the presence of the *mecA* gene, 10 (37%) exhibited resistance to Cefoxitin (30ug). Specifically, 6 of these resistant isolates were obtained from in-patients (22.2%), 2 from outpatients (7%), and 1 (3.7%) each from hospital beds and wastewater. All 10 of the Cefoxitin-resistant strains were subsequently confirmed as MRSA using Oxoid Brilliant Green Agar, resulting in a 100% positive culture rate (Table 2). This study reported an overall MRSA prevalence of 33.7% in the study area. The result slightly exceeds the average prevalence of 30%, as documented by Gorwitz et al. (2006). However, studies conducted in Ilorin (34.7%), Jos (43%), and Benin (79%) reported higher rates than the present study, while studies in Abuja (26.9%), Bauchi (28%), and Kano (28.6%) recorded lower rates (Okon et al. 2014). This increase in prevalence may be associated with various factors, including a rise in the number of outpatients and inpatients due to insurgent activities and a substantial number of internally displaced persons (IDPs) being managed in the facility. Additionally, it may be due to higher antibiotic consumption among hospitalized patients and the significant role that the hospital environment plays in facilitating MRSA spread through patients, healthcare workers, and visitors (Abdullahi and Iregbu 2018). This has negative implications for infection management and underscores the need to promote strict adherence to antibiotic stewardship guidelines and protocols.

**Table 2:** *Staphylococcus aureus* Strains Resistant to Cefoxitin (30 μg)

S/no.	Source of isolates	Resistant isolate	Cefoxitin resistant (%)	Positive growth on oxoid brilliance agar
1	In-Patients	7	6(85.7)	6(85.7)
2	Out-Patients	6	2(33.3)	2(33.3)
3	Hospital Equipment (Bed)	6	1(16.6)	1(16.6)
4	Hospital Wastewater (Sewage)	8	1(12.5)	1(12.5)
	Total	27	10	10

The resistance of MRSA to non-β-lactam antibiotics is a cause for concern as it limits therapeutic options. The rate of MRSA in this study is alarming, although it aligns with antimicrobial susceptibility reported in previous studies; Abdullahi and Iregbu (2018) reported a rate of 68.0%, even higher than the 42.1% recorded in this study. High resistance to commonly used antibiotics such as Amoxicillin, Gentamycin, Ciprofloxacin, Erythromycin, Cefuroxime, and Ceftriaxone is consistent with other studies in Nigeria that have reported high levels of resistance to these drugs (Nwankwo et al. 2010; Motayo et al. 2012; Abdullahi and Iregbu 2018). The presence of insertion sites for plasmids and transposons in the *mecA* complex of MRSA, which often carry antibiotic resistance genes, accounts for resistance to several classes of antibiotics (Abdullahi and Iregbu 2018). Resistance to these drugs is further exacerbated by the irrational prescription of antibiotics and the ready availability of these drugs, which can be purchased over the counter. This, in turn, contributes to the trend of nosocomial infections caused by MRSA and the associated high morbidity and mortality observed in many areas worldwide (Rakette et al. 2012).

**Fig. 1:**
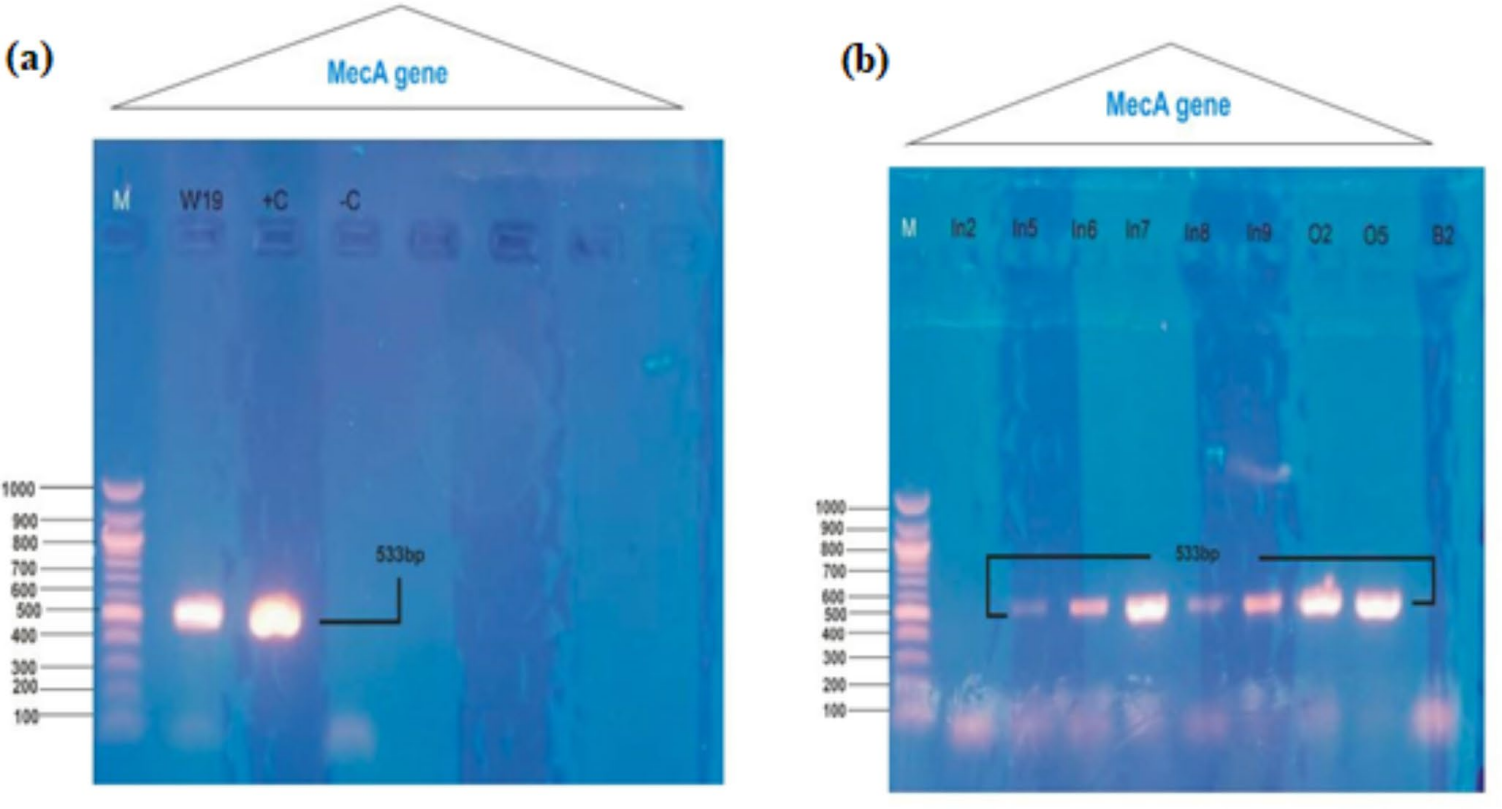
Electrophoresis showing 533 bp DNA fragments corresponding to the *mecA* gene, amplified by PCR. **a** Positive and negative controls for the *mecA* gene, with PCR primers in lanes 8–11, alongside a 1000 bp DNA size marker and a 900 bp molecular weight ladder. **b** Detection of the *mecA* gene in different *S. aureus* isolates

The assay for confirming MRSA yielded a 100% isolation rate on Oxoid Brilliant Agar in this study, affirming the effectiveness of modern methods for detecting *mecA*-carrying *S. aureus* strains using Cefoxitin discs. This increases the reliability and precision of MRSA strain detection (Abubakar and Sulaiman 2018). Studies have shown that most MRSA isolates contain the *mecA* gene, although it may be absent in some MRSA strains. It has also been demonstrated that in addition to the *mecA* gene, *PBP4* and the *icaA* genes cluster can also encode resistance in MRSA (Ogbolu et al. 2011). Genotypically, the resistant *S. aureus* isolates underwent PCR examination for the molecular detection of the *mecA* gene. Figure 1a and b illustrates the distribution pattern of the *mecA* gene among the positive isolates, along with the PCR assay data. Most high-level MRSA isolates carried the *mecA* gene in their genome, resulting in a 533 bp fragment (Fig. 1; lanes 1 to 10). Similar studies in Nigeria have also reported a 533 bp *mecA* gene (Alli et al. 2011; Ibadin et al. 2017).

### Qualitative phytochemical analysis

The phytochemical screening of *A. nilotica* leaves and fruits, as well as *M. indica* leaves, revealed the presence of various bioactive compounds, as shown in Tables 3 and 4. These findings are highly significant because the identified phytochemicals, such as tannins, alkaloids, flavonoids, saponins, and terpenoids, are known for their wide-ranging pharmacological properties, including antimicrobial, antioxidant, and anti-inflammatory activities (Abeysinghe et al. 2021). The detection of these compounds provides a strong foundation for exploring their potential efficacy against *S. aureus*, particularly drug-resistant strains such as MRSA and VRSA.

**Table 3:** *Acacia nilotica* leaves and fruit phytochemical analysis using different solvents

S/no.	Phytochemical constituents	*Acacia nolitica* (Leaves)			*Acacia nolitica* (Fruit)		
		Methanol	Chloroform	Distilled water	Methanol	Chloroform	Distilled water
1	Detection of Flavonoid						
	A. Alkaline Reagent test	–	–	+	–	+	+
	B. Lead acetate test	–	–	+	–	+	+
2	Detection of Tannin						
	A. 5% Ferric chloride	+	+	+	+	+	+
	B. Acetic acid test	+	+	+	+	+	+
	C. Dil. KMnO_4_ Test	+	+	+	+	+	+
3	Detection of Saponins Froth test	–	–	–	+	–	+
4	Detection of Alkaloid						
	A. Wagner’s test	+	+	+	+	+	–
	B. Mayer’s test	+	+	+	+	+	–
5	Detection of cardiac Glycoside Keller-kiliani test	–	–	–	–	–	+
6	Detection of Steroid						
	A. H_2_SO_4_ test	+	–	+	+	–	+
	B. Liebermann-Burchard test	+	–	+	+	–	+
7	Volatile oil test	–	–	–	–	+	+
8	Detection of Terpenoid Salkowski test	+	–	+	–	+	+
9	Detection of Carbohydrates						
	A. Benedict’s test	+	+	+	+	+	+
	B. Fehling’s Test	+	+	+	+	+	+

**Key:** Positive (+) indicates presence, and Negative (–) indicates absence of secondary metabolites**.**

**Table 4:** *Magnifera indica* leaves phytochemical analysis using different solvents

S/no.	Phytochemical Constituents	*Magnifera indica* (Leaves)		
		Methanol	Chloroform	Distilled water
1	Detection of Flavonoid			
	A. Alkaline Reagent test	+	–	+
	B. Lead acetate test	+	–	+
2	Detection of Saponins			
	Froth test	+	–	+
3	Detection of Alkaloid			
	A. Wagner’s test	+	+	+
	B. Mayer’s test	+	+	+
4	Detection of Steroid			
	A.H_2_SO_4_ test	+	–	+
	B. Liebermann-Burchard test	+	–	+
5	Volatile Oil test	–	–	–
6	Detection of Anthraquines Borntrager’s test	+	+	+
7	Detection of Carbohydrates			
	A. Benedict’s test	+	+	+
	B. Fehling’s Test	+	+	+

**Key:** Positive ( +) indicates presence**, and** Negative (-) indicates absence of secondary metabolites**.**

In the *A. nilotica* extracts, methanol, chloroform, and distilled water were used to extract various phytochemical classes. The methanolic leaf extract of *A. nilotica* was rich in tannins, alkaloids, steroids, terpenoids, and carbohydrates. The chloroform extract contained tannins, alkaloids, and carbohydrates, while the distilled water extract contained flavonoids, tannins, alkaloids, steroids, terpenoids, and carbohydrates. This broad range of bioactive compounds correlates with previous studies by Ghanghro et al. (2015) and Saleh et al. (2015), who also reported the presence of these compounds in *A. nilotica*. Detecting tannins in all the extracts is particularly significant because tannins are well-documented for their ability to form complexes with proteins and bacterial cell walls, inhibiting microbial adhesion and enzyme activity (Mamman and Isa, 2013; Bumunang et al. 2020). This property could explain the traditional use of *A. nilotica* for treating infections, as tannins can impede the adhesion of *S. aureus* to host tissues, thereby reducing infection risk. Tannins' ability to precipitate microbial proteins and deprive bacteria of essential nutrients can reduce microbial growth and virulence.

Alkaloids in all *A. nilotica* extracts are known for their antimicrobial properties. Alkaloids have been shown to intercalate into the DNA of pathogens, disrupting the synthesis of nucleic acids and proteins, which is crucial for bacterial growth and replication (Yan et al. 2021). This mechanism of action makes alkaloids particularly effective against Gram-positive bacteria such as *S. aureus*. Given that MRSA and VRSA strains exhibit resistance through altered cell wall synthesis, the ability of alkaloids to interfere with DNA replication could provide a novel mechanism to combat resistant strains. The presence of steroids and terpenoids in the methanolic and distilled water extracts of *A. nilotica* suggests potential anti-inflammatory and antimicrobial activities. Steroids are known to modulate the immune response, which could help reduce inflammation associated with *S. aureus* infections. Terpenoids have also been reported to possess potent antimicrobial properties, particularly against drug-resistant pathogens. The combined presence of terpenoids and steroids could improve the plant's efficacy in reducing infection-related inflammation and microbial load (Masyita et al. 2022).

Similarly, the phytochemical screening of *M.* indica leaves using methanol, chloroform, and distilled water revealed diverse bioactive compounds. The methanolic extract of *M. indica* contained flavonoids, saponins, alkaloids, steroids, anthraquinones, and carbohydrates, while the chloroform extract showed alkaloids, anthraquinones, and carbohydrates. The distilled water extract contained flavonoids, saponins, alkaloids, steroids, anthraquinones, and carbohydrates. These findings are consistent with earlier study by Aiyelaagbe and Osamudiamen (2009), who highlighted the antimicrobial and antioxidant properties of *M. indica* phytochemicals. The presence of flavonoids in both *M. indica* and *A. nilotica* is significant, as flavonoids are known for their broad-spectrum antimicrobial activities. Flavonoids can disrupt microbial membranes, leading to increased permeability and cell death. They also exhibit synergistic effects when combined with other antimicrobial agents, making them suitable candidates for developing new treatments against *S. aureus*, including MRSA strains. Flavonoids' ability to chelate metal ions, essential for bacterial enzymatic processes, further improves their antibacterial potential (Thebti et al. 2023). Saponins, identified in *M. indica* extracts, are known for forming complexes with membrane cholesterol, leading to pore formation and cell lysis in microbes. Saponins also exhibit anti-inflammatory and immune-modulatory properties, which could improve the body's natural defences against *S. aureus* infections (Timilsena et al. 2023). The detection of anthraquinones in methanolic and chloroform extracts of *M. indica* is significant. Anthraquinones possess strong antibacterial properties, often targeting bacterial nucleic acids and proteins. They can inhibit bacterial growth by interfering with DNA gyrase, an essential enzyme for bacterial replication (Qun et al. 2023). This mode of action could make anthraquinones particularly effective against *S. aureus* strains, which rely on rapid replication during infection.

The phytochemical constituents identified in both *A. nilotica* and *M. indica* extracts have profound implications for their potential antimicrobial activity, particularly against *S. aureus*, including its drug-resistant variants such as MRSA and VRSA, which represents a significant challenge in both community and healthcare settings due to its ability to develop resistance to antibiotics. The presence of alkaloids, tannins, flavonoids, and terpenoids in the extracts suggests multiple mechanisms of action against *S. aureus*. Alkaloids and tannins may inhibit bacterial adhesion to host tissues, a critical step in establishing *S. aureus* infections. Flavonoids and terpenoids may disrupt bacterial cell membranes and inhibit vital enzymes required for *S. aureus* survival. Moreover, saponins and anthraquinones may lead to bacterial cell lysis and inhibition of DNA synthesis, effectively reducing bacterial load. Combining these phytochemicals offers a multidimensional approach to combating *S. aureus*, especially considering the pathogen's ability to evade single-drug therapies through resistance mechanisms. The synergy between these compounds could lead to more effective antimicrobial formulations. Furthermore, the antioxidant and anti-inflammatory properties of these phytochemicals can aid in reducing the inflammatory response often associated with *S. aureus* infections, promoting faster recovery and minimizing tissue damage.

The antimicrobial analysis of *Acacia nilotica* and *Mangifera indica* extracts demonstrated varying antibacterial activity against methicillin-resistant *Staphylococcus aureus* (MRSA), as summarized in Table 5. The extracts exhibited promising antibacterial potential compared to the standard antibiotic vancomycin, with the ethanolic extract showing the most potent inhibition across all tested concentrations. This suggests that the bioactive compounds in these extracts, as identified through phytochemical screening, likely contribute to their antibacterial effects. The inhibition zone test revealed that the antibacterial efficacy varied with the extraction solvent, reflecting differences in solubility and extraction efficiency of the phytochemical components. For instance, the chloroform extract of *M. indica* leaves produced a smaller inhibition zone (10.62 mm at 100 mg/mL). In contrast, the ethanolic extract exhibited a significantly larger inhibition zone (22.50 mm), followed by the alcoholic extract (18.27 mm). This trend remained consistent at lower concentrations (75, 50, and 25 mg/mL), indicating that the ethanolic extract consistently showed superior antibacterial activity. This enhanced efficacy can be attributed to the ethanolic extract’s ability to solvate a wide range of polar and non-polar phytochemicals, including tannins, flavonoids, and alkaloids, as confirmed by the phytochemical analysis. Previous studies have demonstrated the antimicrobial potential of these compounds: tannins are known to inhibit bacterial adhesion by forming irreversible complexes with microbial proteins and enzymes, preventing colonization (Isa and Mamman 2013); flavonoids disrupt bacterial cell membranes and inhibit nucleic acid synthesis, making them highly effective against both *S. aureus* and resistant strains like MRSA (Thebti et al. 2023).

**Table 5:** Average zones of inhibition of *M. indica* and *A. nilotica* leaves extracts on MRSA

Concentration (mg/ml)	*M. indica* leaves extract			Van(5 mg/ml)
	CHCl_3_	EtOH	HA	
100	10.62 ± 0.5	22.50 ± 0.27	18.27 ± 0.19	26.00 ± 0.7
75	7.21 ± 0.6	20.25 ± 0.08	18.15 ± 0.13	–
50	5.66 ± 0.2	15.01 ± 0.15	17.00 ± 0.14	–
25	5.20 ± 0.4	10.30 ± 0.50	18.02 ± 0.08	–

Concentration (mg/ml)	*A. nilotica* leaves extracts			Van(5 mg/ml)
	CHCl_3_	EtOH	HA	
100	8.00 ± 1.13	12.50 ± 0.41	5.22 ± 0.66	26.00 ± 0.7
75	6.15 ± 0.5	11.83 ± 0.40	3.23 ± 0.01	–
50	4.50 ± 1.4	10.17 ± 0.15	1.33 ± 0.45	–
25	3.33 ± 0.7	10.12 ± 0.22	1.08 ± 0.02	–

**Key:** HA: alcoholic Van: Vancomycin, CHCL_3_: Chloroform EtOH: ethanol (mm, n = 3).

Remarkably, the *A. nilotica* extracts exhibited a different behaviour in terms of inhibition zone sizes. The alcoholic extract showed a minor inhibition zone (5.22 ± 0.66 mm) at 100 mg/mL. In comparison, the chloroform extract had a moderate zone of inhibition (8.00 ± 1.13 mm), and the ethanolic extract demonstrated the largest zone (12.50 ± 0.41 mm). This observation suggests that ethanol effectively extract more *A. nilotica*'s active phytochemicals, such as alkaloids and terpenoids, aligning with prior reports of these compounds' antibacterial efficacy.

The Minimum Inhibitory Concentration (MIC) and Minimum Bactericidal Concentration (MBC) values provide a quantitative measure of the antimicrobial potency of the extracts. The MIC values ranged between 0 and 6.20 mg/mL, with the ethanolic extract consistently exhibiting the highest MIC values among the tested extracts, indicating its superior inhibitory capability against MRSA (Table 6). The ethanolic extract of *M. indica* leaves had the lowest MIC, suggesting a lower concentration is required to inhibit bacterial growth than the other extracts.

**Table 6:** The MIC and MBC of *M. indica* and *A. nilotica* leaf extracts and *A. nilotica* fruit extracts against the test MRSA

Extract	MIC	MBC
*M. indica* leaf extracts (μg/mL)		
CHCl_3_	2.00	8.2
EtOH	5.40	12.7
HA	3.50	10.3
*A. nilotica* leaf extracts (μg/mL)		
CHCl_3_	1.00	9.2
EtOH	6.20	11.6
HA	3.00	–
*A. nilotica* fruit extracts (μg/mL)		
CHCl_3_	4.00	8.8
EtOH	4.51	10.2
HA	–	–

**Key:** HA, alcoholic; CHCL_3_: Chloroform EtOH: ethanol (mm, n = 3); MIC, Minimum inhibitory concentrations; MBC, Minimum bactericidal concentrations.

The MBC values for the ethanolic extract ranged from 10.2 to 12.7 mg/mL, which were higher than the MIC values, indicating that a higher extract concentration is needed to kill the bacteria outright rather than just inhibit their growth. The MBC values for the chloroform and alcohol extracts ranged between 0 and 10.3 mg/mL. However, the alcoholic extract of *A. nilotica* leaves and fruits was ineffective against MRSA, as evidenced by its higher MBC values, indicating its limited bactericidal potential. These findings suggest that the ethanolic extracts of both *M. indica* and *A. nilotica* are more effective in not only inhibiting but also killing MRSA cells, likely due to the presence of potent bioactive compounds such as phenolic compounds, alkaloids, and flavonoids, which were identified in the qualitative phytochemical analysis. Phenolic compounds have been reported to destabilize bacterial cell membranes and disrupt enzymatic processes, thus leading to bacterial cell death.

The antimicrobial efficacy of the extracts can be directly attributed to the phytochemical constituents identified in the qualitative analysis. Compounds such as tannins, flavonoids, alkaloids, saponins, steroids, and terpenoids in *Acacia nilotica* and *Mangifera indica* extracts are well-known for their antimicrobial properties. They are likely responsible for the observed antibacterial activities. Tannins bind to bacterial proteins and enzymes, disrupting their function and leading to microbial death (Bumunang et al. 2020) in a mechanism that is particularly effective against *S. aureus*, which relies on protein-based adhesions for tissue colonization. Flavonoids exhibit antimicrobial effects by forming complexes with bacterial cell walls, causing structural disintegration and inhibiting nucleic acid synthesis, thus halting bacterial replication. The presence of flavonoids in the ethanolic extracts of both plants correlates with the larger inhibition zones and lower minimum inhibitory concentration (MIC) values observed in the study. Alkaloids, known for their ability to interfere with microbial DNA, further inhibit bacterial growth and replication. This is particularly relevant for MRSA and other resistant strains, which depend on rapid replication for infection. The alkaloids in the ethanolic extracts of *A. nilotica* and *M. indica* could explain their enhanced bactericidal activity, as reflected in the minimum bactericidal concentration (MBC) values. Additionally, both identified in the extracts, saponins, and terpenoids, may further contribute to the antibacterial activity by disrupting microbial cell membranes and inhibiting enzymes critical for energy metabolism. These combined effects highlight the potency of the extracts in combating bacterial pathogens.

The higher antibacterial activity of the ethanolic extracts, as demonstrated by both the larger inhibition zones and lower MIC/MBC values, suggests that ethanol is more effective at extracting these bioactive compounds. This aligns with the general understanding that ethanol is a versatile solvent capable of extracting a broad spectrum of phytochemicals, including polar and non-polar compounds. The observed antibacterial activities, particularly the low MIC values of the ethanolic extracts, underscore the potential of *A. nilotica* and *M. indica* as sources of novel antimicrobial agents for treating infections caused by MRSA. The findings support the hypothesis that these plants contain potent bioactive compounds capable of inhibiting and killing drug-resistant bacteria. These extracts could serve as lead compounds in developing new antimicrobials, particularly for infections resistant to standard antibiotics such as vancomycin. Moreover, the ability of these plant extracts to inhibit MRSA at relatively low concentrations suggests that they could be developed into effective topical or systemic therapies. Their broad range of phytochemical constituents provides a multi-targeted approach to combatting bacterial resistance, which is critical in the fight against multidrug-resistant organisms.

The GC–MS analysis of the ethanolic extracts from *A. nilotica* leaves, fruits, and *M. indica* leaves revealed a rich diversity of bioactive phytoconstituents. A total of 43 distinct compounds were identified by comparing their mass spectra with the National Institute of Standards and Technology (NIST) library. These compounds, characterized by their retention times (RT), molecular formulas, molecular weights, and PubChem IDs, highlight these plant extracts' chemical complexity and potential therapeutic applications (Table 7). For *A. nilotica* leaf extracts, prominent compounds such as α-D-Xylofuranose, cyclic 1,2,3,5-bis(butylboronate), Octadecane, β-Amyrin, and Urs-12-en-3beta-ol were identified. These compounds are known for their wide-ranging biological activities. β-Amyrin is a triterpenoid with reported anti-inflammatory, antimicrobial, and hepatoprotective properties, while Urs-12-en-3beta-ol (ursolic acid) has demonstrated potential as an anticancer and antimicrobial agent (Hernández Vázquez et al. 2012). These bioactive compounds correlate with the antimicrobial activity observed in the phytochemical analysis, particularly against MRSA.

**Table 7:** Chemical Compounds Identified in GC–MS Analyses of Extracts from *A. nilotica* Leaves and Fruits and *M. indica* Leaves Extract

S/no.	Compound	Formula	Molecular Weight	Retention time (min)	PubChem CID
*A. nilotica* leaf extract					
1	α-D-Xylofuranose, cyclic 1,2:3,5-bis(butylboronate)	C_13_H_24_B_2_O_5_	282	17.678	CID_587226
2	Octadecane, 3-ethyl-5-(2-ethylbutyl)-	C_26_H_54_	366	19.281	CID_292285
3	9-(2',2'-Dimethylpropanoilhydrazono)-3,6-dichloro-2,7-bis-[2-(diethylamino)-ethoxy]fluorene	C_30_H_42_Cl_2_N_4_O_3_	576	20.271	CID_590814
4	2-Methyleicosane	C_21_H_44_	296	20.271	CID_519146
5	Desulphosinigrin	C_10_H_17_NO_6_S	279	21.587	CID_9601716
6	Melezitose	C_18_H_32_O_16_	504	21.587	CID_92817
7	Octaethylene glycol monododecyl ether	C_28_H_58_O_9_	538	21.587	CID_20485684
8	β-Amyrin	C_30_H_50_O	426	23.744	CID_73145
9	Dasycarpidan-1-methanol, acetate (ester)	C_20_H_26_N_2_O2	326	30.507	CID_550072
10	Octadecyl 2,2,3-trimethyloctadecaneperoxoate	C_39_H_78_O_3_	594	30.507	CID_53875058
11	3-Hydroxyspirost-8-en-11-one	C_27_H_40_O_4_	428	30.507	CID_628694
12	Nonacosane	C_29_H_60_	408	31.714	CID_12409
13	Urs-12-en-3beta-ol	C_30_H_50_O	426	32.39	CID_225688
14	Heptacosane	C_27_H_56_	380	33.792	CID_11636
15	Carbonic acid, 2-chloroethyl 2-methoxyethyl ester	C_6_H_11_ClO_4_	182	9.244	CID_91692254
*A. nilotica* fruits extract					
16	Melezitose	C_18_H_32_O_16_	504	21.775	CID_92817
17	Maltose	C_12_H_22_O_11_	342	21.775	CID_439186
18	4-O-beta-D-galactopyranosyl-D-glucopyranose	C_12_H_22_O_11_	342	21.775	CID_69301022
19	4,4,6a,6b,8a,11,12,14b-Octamethyl-1,4,4a,5,6,6a,6b,7,8,8a,9,10,11,12,12a,14,14a,14b-octadecahydro-2H-picen-3-one	C_30_H_48_O	424	26.65	CID_124018
20	Octadecane, 3-ethyl-5-(2-ethylbutyl)-	C_26_H_54_	366	30.181	CID_292285
21	1-Monolinolenoyl-rac-glycerol	C_21_H_36_O_4_	352	33.093	CID_5367328
22	8-Methoxy-4-phenylquinoline-2-hydrazine	C_16_H_15_N_3_O	265	33.837	CID_622163
23	1-(3-Fluorophenyl)-2,3,4,9-tetrahydro-1H-beta-carboline	C_17_H_15_FN_2_	266	33.837	CID_201182
24	Carbonic acid, 2-chloroethyl 2-methoxyethyl ester	C_6_H_11_ClO_4_	182	9.187	CID_91692254
25	2,3-Dihydroindole-2-one, 5-methoxy-1,3-dimethyl-3-(dimethylamino)methyl	C_14_H_20_N_2_O_2_	248	9.187	CID_546930
26	Doxepin	C_19_H_21_NO	279	9.187	CID_667477
27	Octadecane, 1,1'-[(1-methyl-1,2-ethanediyl)bis(oxy)]bis-	C_39_H_80_O_2_	580	13.753	CID_545620
28	(22E)-Ergosta-5,22-dien-3-yl acetate	C_30_H_48_O_2_	440	16.997	CID_5352877
29	2-Oxabicyclo[3.3.0]oct-7-en-3-one, 7-(1-hydroxypentyl)-	C_12_H_18_O_3_	210	25.237	CID_592620
30	2-Bromotetradecanoic acid	C_14_H_27_BrO_2_	306	27.360	CID_66340
31	Ethyl 2-bromotetradecanoate	C_16_H_31_BrO_2_	334	27.360	CID_85791
32	Methyl 20-hydroxy-19-oxo-2,16-didehydrocuran-17-oate	C_20_H_22_N_2_O_4_	354	27.972	CID_550468
Mangifera indica leave extract					
33	3-(y-methjlaminopropyl)-5-(4-bromophenyl)-2-methyl-2H-pyrazole	C_14_H_18_BrN_3_	307	9.679	CID_619573
34	N-Methylepinephrine	C_10_H_15_NO_3_	197	11.991	CID_11139
35	Methaqualone	C_16_H_14_N_2_O	250	9.467	CID_6292
36	1,12-Dicarbadodecarboran-2-amine,N-(4-methoxyphenyl)	C_9_H_19_B_10_NO	267	9.679	SID_250105954
37	2-Chloro-8-methoxy-10H-phenothiazine	C_13_H_10_CINOS	263	9.679	CID_621909
38	Methanesulfonanilide, 4'-((3-amino-9-acridinyl)amino)-3'-methoxy-	C_21_H_20_N_4_O_3_S	408	16.946	CID-149559
39	Fluoro-2-nitroaniline,5-[4-(pyrrolidin-1-yl)carbonylmethylpiperazin-1-yl]	C_16_H_22_FN_5_O_3_	351	16.946	CID_620007
40	(22E)-Ergosta-5,22-dien-3-yl acetate	C_30_H_48_O_2_	440	19.755	CID_5352877
41	N-Cyclohexyl-4-methylbenzenesulfonamide	C_13_H_19_NO_2_S	253	19.755	CID_6633
42	1-[4-(Triisopropylsilyl)oxylp henyl]-2-(4-hydroxy-4-pheny1piperidino)-1-propanone	C_29_H_43_NO_3_Si	481	20.597	CID_10254642
43	5-methylsalicylic acid	C_8_H_8_O_3_	152	25.895	CID_6973

**Fig. 2:**
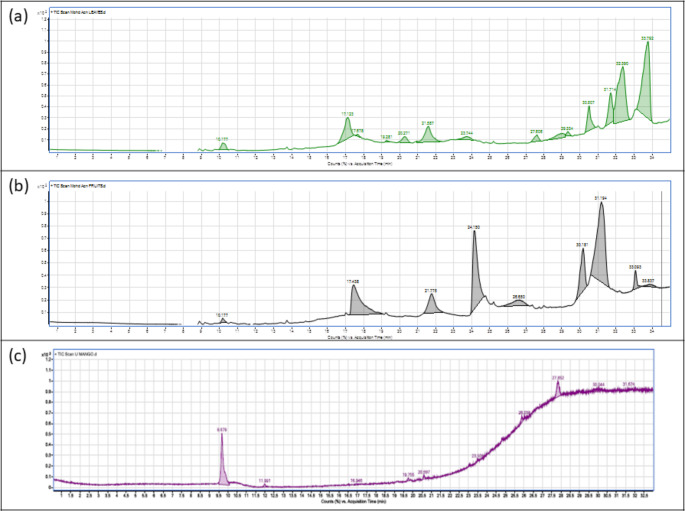
GC–MS Chromatogram **a**  *A. nilotica* leaves extract, **b**  *A. nilotica* fruits extract, **c**  *M. indica* leaves Extract

The *A. nilotica* fruit extracts also contained a variety of bioactive compounds, including Melezitose, Maltose, Octadecane, and Doxepin. Doxepin, a well-known tricyclic antidepressant, has also shown some degree of antimicrobial activity, further supporting the antibacterial effects observed in the study (Hua et al. 2013). Moreover, the identification of Octadecane, a long-chain hydrocarbon, suggests a potential role in membrane disruption of bacterial cells, contributing to the observed antimicrobial efficacy. The *M. indica* leaf extracts identified bioactive compounds such as N-Methylepinephrine, Methaqualone, 2-chloro-8-methoxy-10 h-phenothiazine, and 5-methylsalicylic acid. N-methylepinephrine, a derivative of epinephrine, is significant for its potential cardiovascular effects but also holds antimicrobial properties. Methaqualone, traditionally used as a sedative, has been identified to have potential antimicrobial effects against several bacterial strains. 5-methyl salicylic acid, a known antioxidant and anti-inflammatory agent, also supports the antimicrobial efficacy of *M. indica* extracts. The identification of compounds such as 2-chloro-8-methoxy-10H-phenothiazine is particularly important due to the antimicrobial activity of phenothiazines against various bacterial strains, including multi-drug-resistant *S. aureus*. This further correlates with the extracts' observed inhibition zones and minimum inhibitory concentrations (MIC) when tested against MRSA. The observed antibacterial activity of the plant extracts against MRSA, as demonstrated in the antimicrobial assay and minimum bactericidal concentration (MBC) tests, can be directly linked to the bioactive compounds identified in the GC–MS analysis. The combined action of these phytoconstituents, particularly the presence of compounds with known antimicrobial properties such as β-Amyrin, ursolic acid, N-Methylepinephrine, and phenothiazines, suggests that these extracts hold significant potential as sources of novel antibacterial agents, particularly for drug-resistant pathogens like MRSA. The GC–MS spectrum profile further confirmed the presence of these 43 major components (Fig. 2a–c and Table 7). These identified compounds were focus of further studies.

The GC–MS analysis of the ethanolic extracts of *A. nilotica* leaves and fruits, as well as *M. indica* leaves, provided a valuable understanding of these plant extracts' chemical composition and potential therapeutic applications. The identification of 43 phytoconstituents highlights the complexity and richness of these extracts. A detailed analysis of these compounds' physicochemical and medicinal chemistry properties was conducted to assess their suitability for drug development, adhering to widely recognized drug-likeness rules.

The compounds identified from *A. nilotica* and *M. indica* were subjected to drug-likeness screening, which involved evaluating their molecular weight, hydrogen bond donors (HBD), hydrogen bond acceptors (HBA), lipophilicity (LogP), and topological polar surface area (TPSA), among other parameters (Table 8). These properties are critical in predicting the compounds' bioavailability, permeability, and overall drug-likeness, which are essential in drug design and discovery. Lipinski's Rule of Five is a crucial criterion in drug design, particularly for assessing the potential of compounds to be orally bioavailable. According to Lipinski et al. (1997), an ideal drug candidate should have a molecular weight of ≤ 500 Da, LogP ≤ 5, no more than 5 hydrogen bond donors, and no more than 10 hydrogen bond acceptors. These parameters directly relate to a compound's ability to pass through biological membranes, making them crucial in drug discovery.

**Table 8:** Physicochemical and Medicinal Properties of the Phytochemicals derived from the three extracts

S/no.	Compound CID	Molecular weight	No. of HBA	No. of HBD	MLogP	WLogP	No. of Atom	Molar Refractivity	No. Rotatable bonds	TPSA (Å^2^)	PAINS
*Acacia nilotica* leaves extract											
1	CID_587226	282	5	0	0.21	2.12	44	77.31	6	46.15	0 alerts
2	CID_292285	366	0	0	8.66	9.96	80	127.10	20	0.00	0 alerts
3	CID_590814	576	7	7	3.87	6.33	81	162.22	15	66.40	0 alerts
4	CID_519146	296	0	0	7.60	8.29	66	103.06	17	0.00	0 alerts
5	CID_9601716	279	7	5	–2.09	–1.12	35	65.33	5	148.04	0 alerts
6	CID_92817	504	16	11	–6.15	–7.57	66	100.54	8	268.68	0 alerts
7	CID_20485684	538	9	1	0.41	4.02	95	146.56	26	94.07	0 alerts
8	CID_73145	426	1	1	6.92	8.17	81	134.88	0	20.23	0 alerts
9	**CID_550072**	326	**4**	**1**	**2.82**	**3.14**	**50**	**100.17**	**4**	**45.33**	0 alerts
10	CID_53875058	594	3	0	8.77	13.87	120	191.70	35	35.53	0 alerts
**11**	**CID_628694**	428	**4**	**1**	**3.98**	**5.04**	**71**	**121.79**	**0**	**55.76**	0 alerts
12	CID_12409	408	0	0	9.25	11.56	89	141.52	26	0.00	0 alerts
**13**	**CID_225688**	426	**1**	**1**	6.92	8.02	81	135.14	0	20.23	0 alerts
14	CID_11636	380	0	0	8.86	10.78	83	131.90	24	0.00	0 alerts
**15**	CID_91692254	182	4	0	0.30	1.02	22	39.60	7	44.76	0 alerts
*Acacia nilotica* fruits extract											
16	CID_92817	504	16	11	–6.50	–7.57	66	100.54	8	268.68	0 alerts
17	CID_439186	342	11	8	–4.37	–5.40	47	68.12	4	189.53	0 alerts
18	CID_69301022	342	11	8	–4.37	–5.40	47	68.12	4	189.53	0 alerts
19	CID_124018	424	1	0	6.82	8.23	79	134.18	0	17.07	0 alerts
20	CID_292285	366	0	0	8.66	9.96	80	127.10	20	0.00	0 alerts
21	CID_5367328	352	4	2	3.33	4.47	61	105.25	17	66.76	0 alerts
22	CID_622163	265	4	2	2.62	3.01	35	80.88	3	60.17	0 alerts
23	CID_201182	266	2	2	3.27	3.26	35	82.09	1	27.82	1 alerts
24	CID_91692254	182	4	0	0.30	1.02	22	39.60	7	44.76	0 alerts
25	CID_546930	248	4	0	1.40	1.11	28	75.14	3	32.78	0 alerts
26	CID_667477	279	2	0	3.43	3.81	42	87.68	3	12.47	0 alerts
27	CID_545620	580	2	0	8.17	13.93	121	191.76	37	18.46	0 alerts
28	CID_5352877	440	2	0	6.61	7.98	80	137.69	6	26.30	0 alerts
29	**CID_592620**	210	**3**	**1**	**1.77**	**1.80**	**33**	**57.54**	**4**	**46.53**	0 alerts
30	CID_66340	306	2	1	4.07	5.15	44	79.05	12	37.30	0 alerts
31	CID_85791	334	2	0	4.56	5.62	50	88.18	14	26.30	0 alerts
32	CID_550468	354	6	2	1.17	0.25	48	101.78	3	78.87	0 alerts
*Mangifera indica* leaves extract											
33	**CID_619,573**	307	**3**	**1**	**2.64**	**3.00**	**36**	**78.81**	**5**	**29.85**	0 alerts
34	**CID _11139**	197	**4**	**3**	**0.37**	**0.37**	**28**	**53.93**	**3**	**63.93**	1 alerts
35	CID_6292	250	3	0	2.99	3.00	33	77.27	1	34.89	0 alerts
36	SID_250105954	267	2	1	1.51	–1.94	40	158.67	43	64.23	0 alerts
37	CID -621,909	263	2	1	3.63	4.18	28	74.44	1	46.56	0 alerts
38	CID_149559	408	7	3	1.68	4.99	49	117.96	5	114.72	1 alerts
39	**CID_620007**	351	**7**	**1**	**0.53**	**0.35**	**47**	**104.82**	**5**	**98.63**	0 alerts
40	CID_5352877	440	2	0	6.61	7.98	80	137.69	6	26.30	0 alerts
41	**CID_6633**	253	**3**	**1**	**2.04**	**3.69**	**36**	**69.22**	**3**	**54.55**	0 alerts
42	CID _10254642	481	4	1	3.80	6.31	77	148.52	9	49.77	0 alerts
43	CID_6973	152	3	2	1.32	1.40	19	40.39	1	57.53	0 alerts

**Key:** No. of HBD = hydrogen bond donors, No. of HBA = hydrogen bond acceptors, WLogP = Water–Oil Partition Coefficient, MLogP = Molecular Logarithm of the Partition Coefficient, TPSA = topological polar surface area and PAINS = *Pan*-Assay Interference Structural.

Significant are in value [bold].

In this study, several compounds from *A. nilotica* and *M. indica* failed to meet these criteria. These include seven compounds from *A. nilotica* leaf extract (CID_292285, CID_590814, CID_519146, CID_92817, CID_53875058, CID_12409, and CID_11636) and five from the *A. nilotica* fruit extract (CID_92817, CID_439186, CID_69301022, CID_292285, and CID_545620) were excluded due to their non-compliance with more than one aspect of Lipinski's rule. The failure of these compounds to meet the rule suggests that they may face challenges in oral bioavailability, limiting their potential as therapeutic agents unless modified for improved drug-likeness. Egan's rule emphasizes the importance of lipophilicity (WLogP ≤ 5.88) and TPSA (≤ 131 Å) for predicting oral bioavailability. Similarly, Veber's rule focuses on the number of rotatable bonds (≤ 10) and TPSA (≤ 140 Å) to predict oral drug absorption (Veber et al. 2002). Compounds that failed to meet more than one of these rules were excluded from further analysis. Three compounds from the *A. nilotica* leaf extract (CID_20485684, CID_73145, and CID_225688) and three from the *A. nilotica* fruit extract (CID_124018, CID_5352877, and CID_85791) failed to comply with Egan and Veber's rules. One compound from *M. indica* (SID_250105954) did not meet these criteria (Table 8). This failure indicates that these compounds might have low oral bioavailability, affecting their therapeutic potential in drug development.

Of the 43 initially identified compounds, only 22 met the essential physicochemical and medicinal chemistry criteria for further investigation (Table 8). These compounds exhibit properties that align with the fundamental principles of drug design, such as optimal molecular weight, adequate lipophilicity, appropriate hydrogen bond donors and acceptors, and favourable TPSA. The compounds that pass these criteria are more likely to have good oral bioavailability, essential for developing oral therapeutic agents. Identifying drug-like compounds by applying Lipinski's, Egan's, and Veber's rules provide a foundation for further drug development. The compounds that passed the drug-likeness criteria can now be subjected to more advanced computational analyses, such as molecular docking and molecular dynamics simulations, to predict their binding affinity to bacterial targets. Given the rise of antibiotic-resistant bacteria such as MRSA, discovering natural compounds with drug-like properties offers a promising avenue for developing new antibacterial therapies. The presence of bioactive compounds in *A. nilotica* and *M. indica* suggests that these plants could serve as valuable resources in the search for alternative antimicrobial agents, particularly for addressing drug resistance.

The pharmacokinetic analysis of the bioactive compounds identified in the extracts of *A. nilotica* and *M. indica* is crucial in determining their viability as drug candidates. The fundamental pharmacokinetic properties analyzed include absorption, distribution, metabolism, excretion, and toxicity (ADMET), essential for evaluating the compounds' effectiveness and safety in vivo. These properties provide an understanding of how the compounds behave in the human body and their potential side effects, guiding the decision-making process in drug design and discovery. The ability of a compound to be absorbed through the human intestinal tract and pass-through biological barriers such as the blood–brain barrier (BBB) is critical for its effectiveness as a drug, particularly for orally administered drugs. This study predicted ADMET properties using the AdmetSAR tool (Cheng et al. 2012) and the DataWarrior tool (Sander et al. 2015). These tools allowed for predicting Human Intestinal Absorption (HIA) and BBB permeation, vital for assessing oral bioavailability and central nervous system activity.

The results showed that, except CID_9601716, all the selected compounds were predicted to be effectively absorbed in the human intestine. This suggests that most compounds have favourable oral bioavailability, aligning with the results from the physicochemical analysis, which showed that most compounds adhered to Lipinski's Rule of Five (Lipinski et al. 1997). The prediction that most compounds pass the HIA threshold indicates their potential as orally active agents. Furthermore, the ability of compounds to cross the BBB is particularly relevant for drugs targeting the central nervous system (CNS) or infections affecting the brain, such as bacterial meningitis. In this analysis, all the compounds except CID_11139 were predicted to cross the BBB. This characteristic increases their therapeutic potential for CNS-related conditions, broadening the scope of their applicability in drug development. However, the ability to permeate the BBB must be balanced with safety, as CNS-active drugs may pose risks of neurotoxicity (Table 9).

**Table 9:** Pharmacokinetics properties of the selected phytochemicals derived from the three extracts

S/no.	Compound CID	BBB	HIA	CYP450 2D6 Inhibitor	CYP3A4 Inhibitor	Ames Toxicity	Carcinogens	Acute Oral Toxicity	Mutagenic	Tumorigenic	Reproductive Effect	Irritant
	*A. nilotica* leave extract											
1	CID_587226	+	+	None	None	None	None	III	No	No	No	Low
5	CID_9601716	+	–	None	None	None	None	III	No	No	No	No
9	CID_550072	+	+	Inhibitor	None	None	None	III	No	No	No	No
11	CID_628694	+	+	None	None	None	None	III	No	No	No	No
15	CID_91692254	+	+	None	None	None	None	III	Low	High	High	High
	*A. nilotica* fruits extract											
21	CID_5367328	+	+	None	None	None	None	IV	No	No	No	No
22	CID_622163	+	+	None	Inhibitor	Toxic	None	III	No	High	No	No
23	CID_201182	+	+	Inhibitor	Inhibitor	None	None	III	Low	No	No	No
24	CID_91692254	+	+	None	None	None	None	III	Low	High	High	High
25	CID_546930	+	+	None	None	None	None	III	No	No	No	No
26	CID_667477	+	+	Inhibitor	None	None	None	II	No	No	High	No
29	CID_592620	+	+	None	None	None	None	III	No	No	No	No
30	CID_66340	+	+	None	None	None	None	III	High	No	High	No
32	CID_550468	–	+	None	None	None	None	III	No	No	High	No
	*M. indica* leaf extract											
33	CID_619,573	+	+	None	None	None	None	III	No	No	No	No
34	CID _11139	–	+	None	None	None	None	III	No	No	No	No
35	CID_6292	+	+	None	None	Toxic	None	II	No	No	High	No
37	CID -621,909	+	+	None	Inhibitor	None	None	III	No	No	High	No
38	CID_149559	+	+	None	Inhibitor	Toxic	None	III	High	High	Low	No
39	CID_620007	+	+	None	None	None	None	III	No	No	No	No
41	CID_6633	+	+	None	None	None	None	III	No	Low	High	No
43	CID_6973	+	+	None	None	None	None	III	High	No	No	No

Cytochrome P450 (CYP450) enzyme inhibition is another critical factor in drug metabolism, as it can lead to drug-drug interactions and adverse side effects. Most of the compounds in this study were predicted to be non-inhibitors of CYP450 2D6, which minimizes the risk of metabolic interactions and improves the safety profile of these compounds for therapeutic use. However, a few compounds, including CID_550072, CID_201182, and CID_667477 were identified as CYP450 inhibitors, indicating their potential for adverse interactions with other drugs metabolized by the same enzyme. These compounds may require structural modification or combination therapy to mitigate this risk (Table 9). Similarly, CYP3A4 is another enzyme involved in drug metabolism, particularly in the many commonly used drugs (e.g., statins, corticosteroids, and immunosuppressants) and in the activation or inactivation of numerous xenobiotics. Although five compounds (CID_622163, CID_149559, CID_201182 and CID-621909) were found to inhibit such enzyme.

Toxicity is a primary concern in drug development, as even highly effective compounds may be unsuitable for use due to adverse effects. The Ames test for mutagenicity is one of the standard toxicity tests used to predict whether a compound has the potential to cause genetic mutations, which could lead to cancer or other health issues. In this study, three compounds (CID_622163, CID_667477, and CID_201182) were predicted to be Ames toxic, while four other compounds (CID_6184, CID_454, CID_612550, and CID_6058) showed varying degrees of mutagenic potential (Table 9). Identifying mutagenic or Ames toxic compounds is critical, signalling potential carcinogenicity or genetic damage. This highlights the need for caution when considering these compounds as drug candidates. CID_5367328 was identified as falling into Category IV for acute oral toxicity, with a lethal dose (LD_50_) value exceeding 5000 mg/kg, making it unsuitable for drug development. In contrast, compounds with low toxicity and acceptable safety margins can be further optimized for therapeutic use (Table 9).

The LD_50_ values were categorized according to the US EPA criteria, with Category I representing compounds with highly toxic profiles (LD_50_ ≤ 50 mg/kg) and Category IV representing compounds with the lowest toxicity (LD_50_ > 5000 mg/kg). Most of the compounds in this study fell within Categories II to IV, suggesting moderate to low acute toxicity, which is a favourable outcome for drug development. However, one compound, CID_5367328, was found to have high toxicity, excluding it from further consideration (Table 9).

Tumorigenicity and reproductive toxicity are also essential factors in determining the long-term safety of a drug. Five compounds (CID_91692254, CID_622163, CID_91692254, CID_149559, and CID_6973) were predicted to have tumorigenic potential, and six compounds (CID_91692254, CID_201182, CID_91692254, CID_66340, CID_149559, and CID_6973) were predicted to be mutagenic (Table 9).

The pharmacokinetic properties observed in this study align closely with the physicochemical analyses, reinforcing those drug-likeness criteria such as molecular weight, LogP, hydrogen bond donors/acceptors, and TPSA to predict a compound's behaviour in biological systems. The compounds that passed Lipinski's Rule of Five and other drug-likeness rules generally exhibited favourable pharmacokinetic profiles, particularly in absorption, metabolism, and low toxicity. This correlation emphasizes the importance of an integrated approach in drug design, where physicochemical properties are evaluated alongside pharmacokinetic attributes.

The findings from this pharmacokinetic analysis provide a vital understanding of the potential for these compounds to be developed into therapeutic agents. While most of the selected compounds exhibit promising pharmacokinetic profiles, including high oral bioavailability, BBB permeability, and low toxicity, a few compounds were identified as having undesirable properties, such as mutagenicity, tumorigenicity, and CYP450 inhibition (Table 9).

In drug design, compounds with favourable ADMET properties are more likely to progress through the development pipeline and become viable drug candidates. Excluding compounds with poor pharmacokinetic profiles ensures that only the most promising candidates are considered for further optimization and clinical trials. This step is crucial in minimizing the risk of failure in later stages of drug development.

Structural analysis of PBP2a from MRSA revealed its three distinct domains: Domain I (NTF2-like N-terminal transpeptidase, residues 26-140), Domain II (Penicillin-binding protein dimerization, residues 148-309), and Domain III (Penicillin-binding protein transpeptidase, residues 346-657). Inhibiting these domains, especially Domain III, would disrupt the enzyme's activity. Molecular docking of the five selected compounds against PBP2a from MRSA revealed diverse binding interactions, including hydrogen bonding, hydrophobic interactions, and van der Waals forces, contributing to their binding stability. The free binding energies of the compounds ranged from -5.95 to -10.46 kcal/mol, indicating varying affinities for the target active site of PBP2a (Table 10). CID_628694 exhibited the lowest binding energy (-10.46 kcal/mol), forming hydrogen bonds with Gln521 and interacting hydrophobically with Tyr344, Lys394, Leu603, and Ile614. These interactions occur within Domain III (Penicillin-binding protein transpeptidase), which is crucial for the enzyme's catalytic function. The favourable interactions between CID_628694 and key residues suggest that this compound could effectively inhibit PBP2a, potentially leading to impaired cell wall synthesis in MRSA.

**Table 10:** Molecular docking analysis of the selected compounds against PBP2a from MRSA

S/no.	Compound	Docking scores (Kcal/mol)	H-bond	Distance	Hydrophobic interactions	van der Waals Interactions
1	CID_628694	–10.46	Gln521	2.63	Tyr344, Lys394, Leu603 and Ile614	Ser400, Glu602, Lys604, Gln521, Thr399, Gly345, Asn632 and Lys634
2	CID_546930	–7.60	Trp616	2.68	Ala601, Leu603, Ile614 and Asn632	Thr399, Gly402, Gln521, Glu602 and Lys604
			Ser400	4.87		
3	CID_592620	–6.99	Trp616	2.76	Ala601, Glu602, Leu603 and Il614	Thr399, Pro401, Gly402, Ser403, Gln521, Gly599, Thr600, Gly615 and Asn632
			Ser400	2.67		
			Lys604	3.29		
4	CID_619573	–5.95	Thr600	2.91	Tyr446, Gly599 and Ala642	Glu447, Ser461 and Asn464
			Thr600	2.71		
			Thr600	3.35		
			Ser403	2.70		
			Lys406	2.62		
			Ser462	2.66		
			Ser462	2.54		
5	CID_620007	–7.25	Thr399	3.06	Gln521 and Gly522	Tyr344, Ser400, Lys604, Leu603, Ile614, Trp616 and Lys634
			Asn632	2.85		

Other compounds, such as CID_546930 (-7.60 kcal/mol) and CID_592620 (-6.99 kcal/mol), also demonstrated promising binding affinities, with hydrogen bonds formed with Trp616, Ser400, and Lys604, alongside substantial hydrophobic and van der Waals interactions. These residues are essential for stabilizing the active site of PBP2a, reinforcing the potential of these compounds as competitive inhibitors. CID_619573 (-5.95 kcal/mol) showed the lowest binding affinity, though it formed the highest number of hydrogen bonds (seven) with residues such as Thr600, Ser403, Lys406, Ser462, and others in Domain III. This suggests that even compounds with moderate binding energies can achieve significant inhibitory effects if they engage in multiple interactions critical for enzymatic activity. The differences in docking scores and interaction profiles can be attributed to the varying chemical structures of the phytochemicals. Compounds with polar functional groups (e.g., hydroxyl or amine groups) tend to form more hydrogen bonds, stabilizing their interaction within the active site. The hydrophobic portions of the molecules contribute to van der Waals and hydrophobic interactions, which are essential for binding in the non-polar regions of the PBP2a active site. The high activity of CID_628694, as demonstrated by its low binding energy may be due to an optimal balance between hydrophilic and hydrophobic regions, allowing it to establish both hydrogen bonds and hydrophobic contacts, thus providing more stable binding. In contrast, CID_619573's weaker binding affinity, despite forming several hydrogen bonds, could be related to suboptimal spatial orientation or less effective van der Waals interactions with non-polar residues. Furthermore, previous structural studies have highlighted the importance of Domain III in PBP2a as the critical catalytic site (Ambade et al. 2023). The ability of the selected compounds to interact with residues in this domain, particularly Gln521, Lys604, and Leu603, suggests that these compounds target the enzyme's most vulnerable region. This is consistent with findings from other studies that emphasize the importance of inhibiting Domain III to block the transpeptidase activity of PBP2a and prevent the cross-linking of peptidoglycan, which is crucial for bacterial survival under beta-lactam stress (Nauta et al. 2021).

### Molecular dynamics simulation analysis

**Fig. 3:**
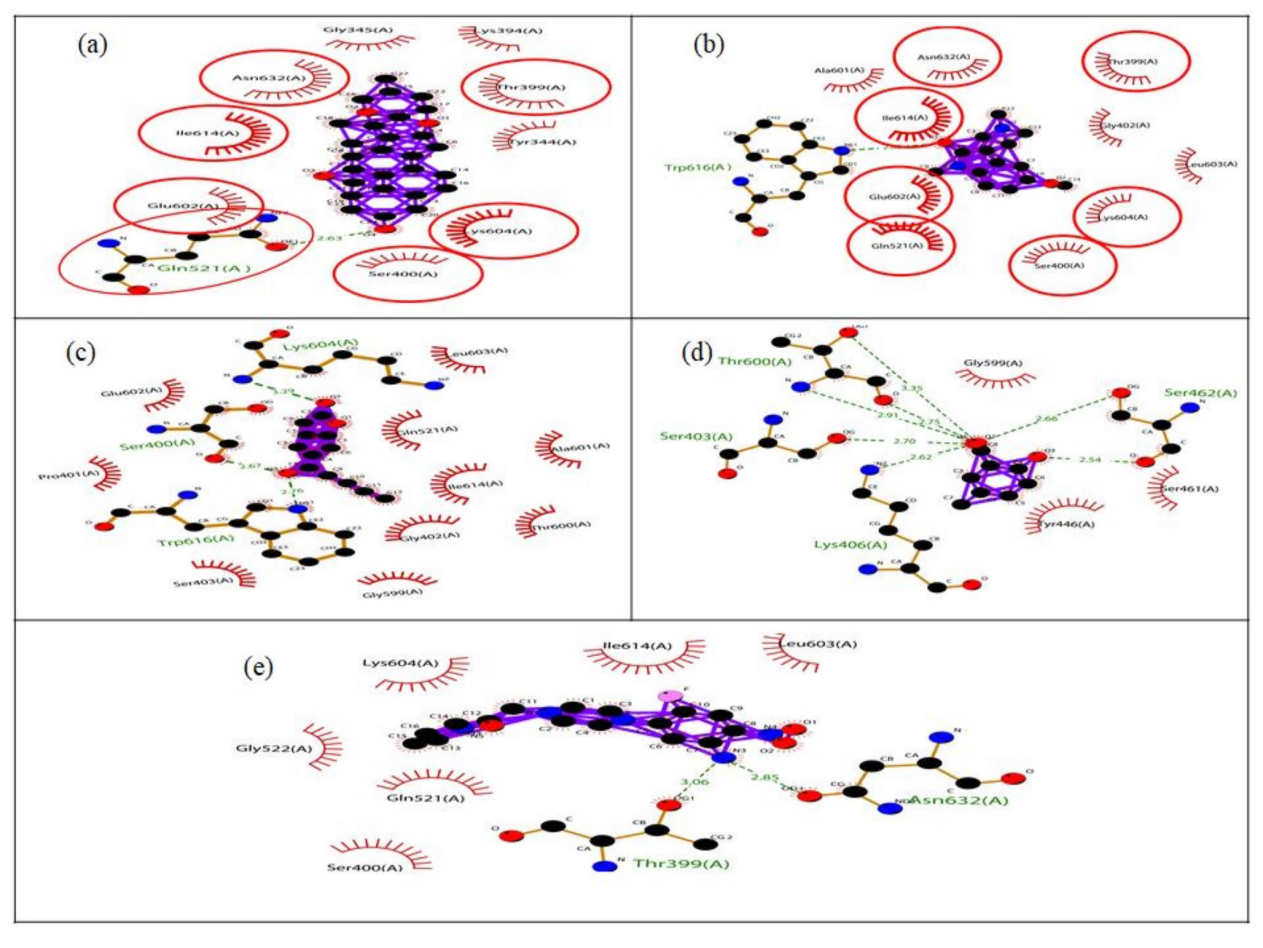
The 2D interactions between the chosen compounds and PBP2a reveal the presence of hydrogen bonds and hydrophobic interactions, **a** CID_628694, **b** CID_546930, **c** CID_592620, **d** CID_619573, **e** CID_620007

**Fig. 4:**
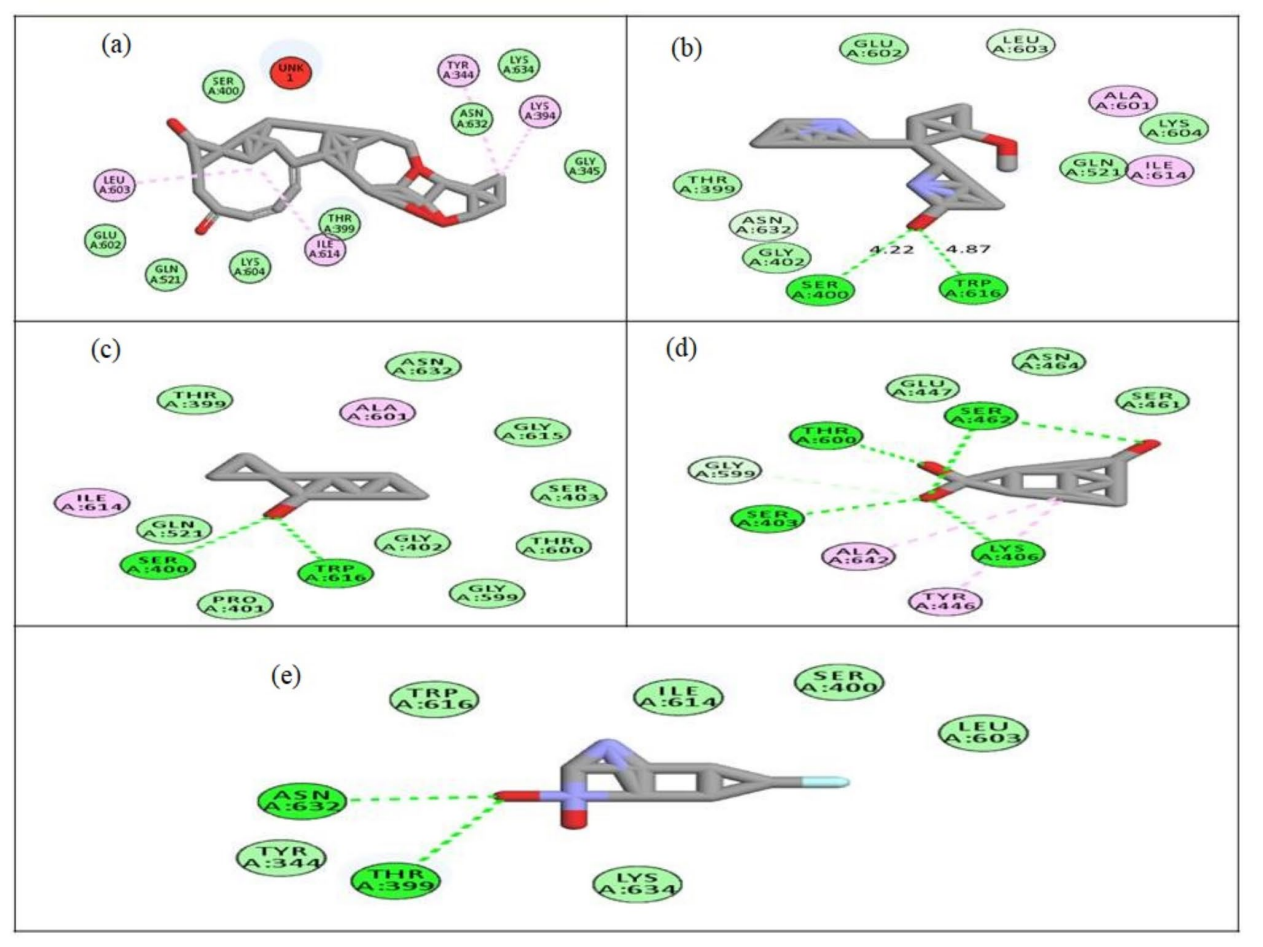
The selected compounds interact in two dimensions with PBP2a, involving the formation of hydrogen bonds, van der Waals forces, and hydrophobic interactions, **a** CID_628694, **b** CID_546930, **c** CID_592620, **d** CID_619573, **e** CID_6200. **Key: Green line**: Indicating Hydrogen bond while pink line indicating hydrophobic interactions

**Fig. 5:**
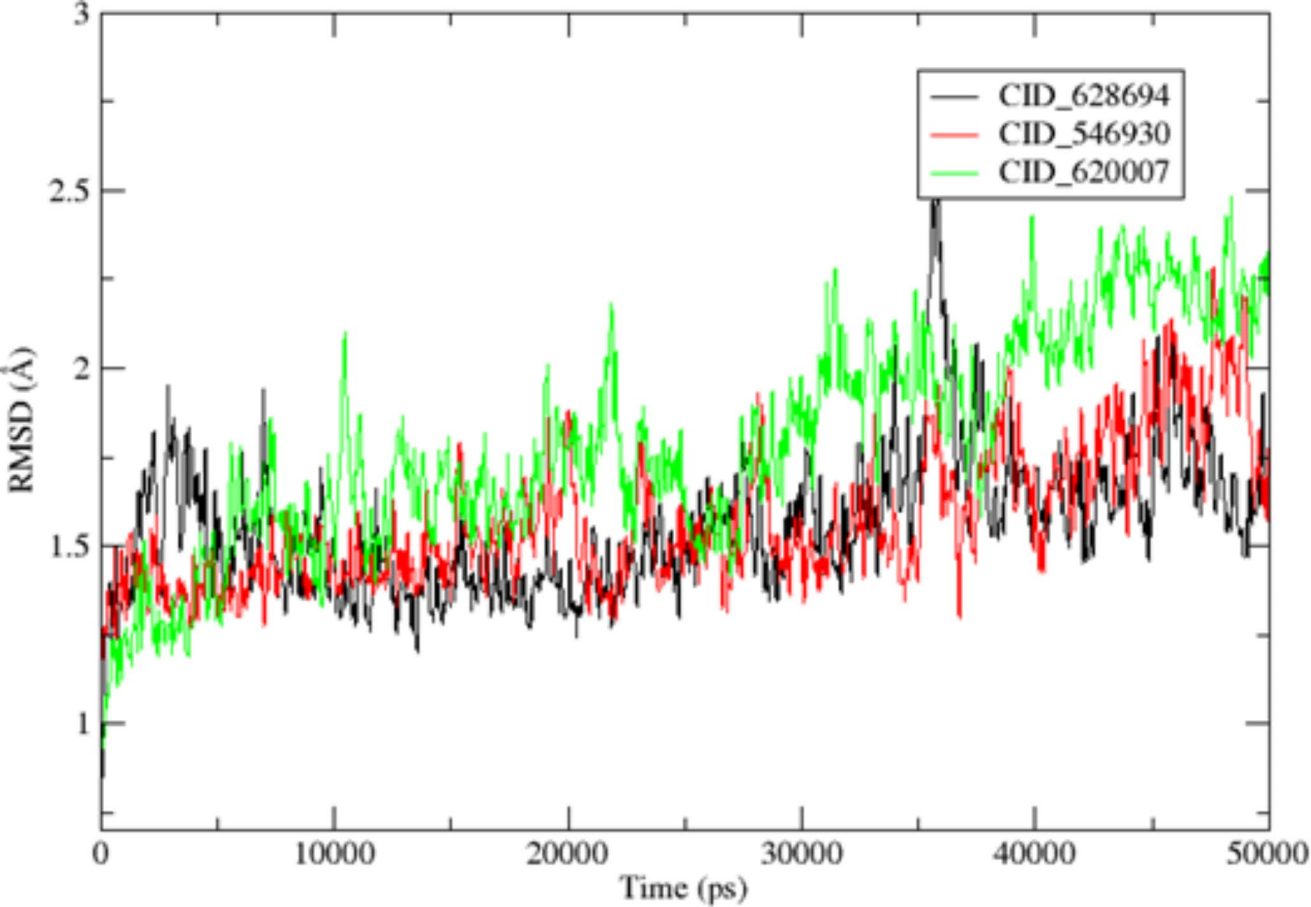
The Root Mean Square Deviation (RMSD) values for PBP2a when bound to the chosen ligands—specifically, PBP2a─CID_628694, PBP2a─CID_546930, and PBP2a─CID_620007

Molecular Dynamics (MD) simulation was performed on three selected PBP2a-ligand complexes (PBP2a–CID_628694, PBP2a–CID_546930, and PBP2a–CID_620007), which demonstrated the most favourable binding energies in docking analysis (-10.46 kcal/mol, -7.60 kcal/mol, and -7.25 kcal/mol, respectively). The objective was to assess the stability and dynamic behaviour of these complexes over 50 ns and to validate the results obtained from docking studies by observing how these ligands interact with PBP2a in a dynamic system. RMSD analysis is crucial in assessing protein–ligand complexes' structural stability and conformational shifts over time (Figs. 3, 4). The low RMSD values for the three complexes indicate that the ligands remained stably bound to the active site of PBP2a throughout the simulation. PBP2a–CID_628694 complex reached equilibrium within 10 ns, with an average RMSD of 1.5592 ± 0.00613 Å, indicating minimal conformational changes (Fig. 5). The relatively low maximum RMSD (2.48 Å) suggests that the ligand maintained solid and stable interactions with the protein, particularly with the flexible loop regions, which are vital for enzymatic function. This stability correlates well with the docking results, where CID_628694 exhibited the lowest binding energy and formed strong hydrogen bonds with critical residues like Gln521 (Fig. 5). The MD simulation reinforces that CID_628694 can strongly inhibit PBP2a through stable interactions.

Similarly, PBP2a–CID_546930 exhibited the same pattern, with equilibrium achieved after 3ns and a stable RMSD of 1.5744 ± 0.00615 Å. The low fluctuations in this complex indicate that the ligand's interactions with the active site remained intact, confirming its inhibitory solid potential as predicted by the docking results (Fig. 5). The consistency between docking and MD simulation suggests that CID_546930 forms stable, long-lasting interactions, particularly in the flexible loop regions, enhancing its potential as an effective PBP2a inhibitor. Similarly, the PBP2a–CID_620007 complex displayed slightly higher RMSD values (average 1.7963 ± 0.00962 Å), indicating greater flexibility. However, the complex remained stable within the RMSD range with minimal deviations. This observation suggests that CID_620007 forms a stable complex with PBP2a in a dynamic system despite the moderate binding energy obtained from docking. These findings indicated that the ligands with unfavourable docking scores can still exhibit stability during MD simulations. This shows the importance of combining the docking and MD simulation analyses in drug design, as shown in Fig. 5.

**Fig. 6:**
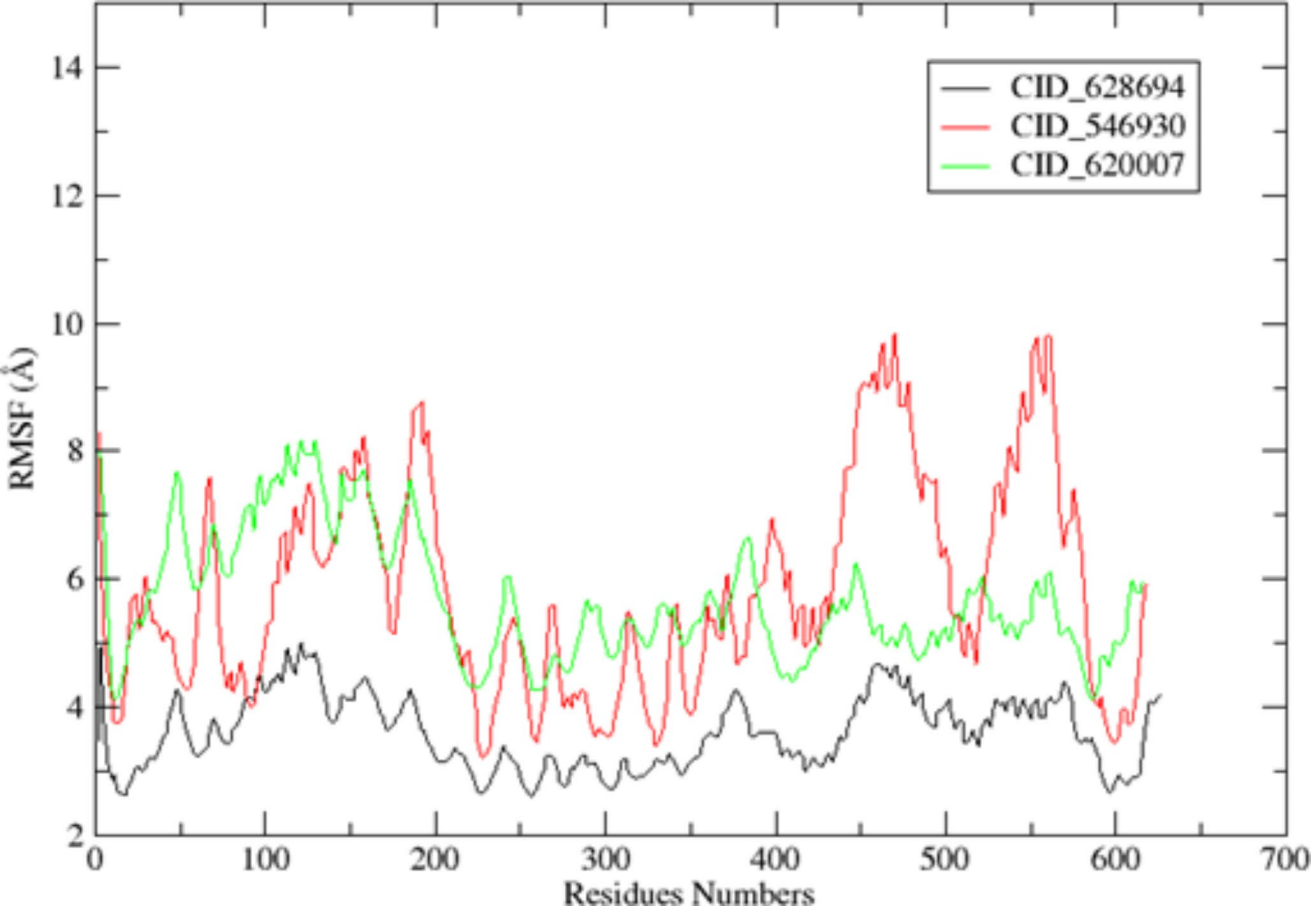
The Root Mean Square Fluctuation (RMSF) values for PBP2a when bound to the chosen ligands—specifically, PBP2a─CID_628694, PBP2a─CID_546930, and PBP2a─CID_620007

RMSF analysis provided an understanding of the mobility of individual amino acids during the simulation. Most residues displayed low RMSF values for all three complexes, particularly those in the flexible loop regions. These areas are typically more dynamic, but in the presence of the ligands, they became more rigid, suggesting that the compounds effectively stabilized these protein regions. Mainly, residues such as Gln521, Lys604, and Leu603, crucial for PBP2a's catalytic function, exhibited minimal fluctuations due to consistent interactions with the ligands. This further supports the findings from the docking study, where these residues were identified as vital interaction points. The RMSF results highlight that the ligands bind strongly to PBP2a and reduce the mobility of essential regions, thereby augmenting the inhibitory effect (Fig. 6). Higher RMSF values were observed in protein regions not directly involved in binding the ligands. These regions, being less critical to enzymatic function, do not impact the overall stability of the protein-ligand complex, allowing the ligands to exert their inhibitory effects without disrupting non-essential areas of the protein.

**Fig. 7:**
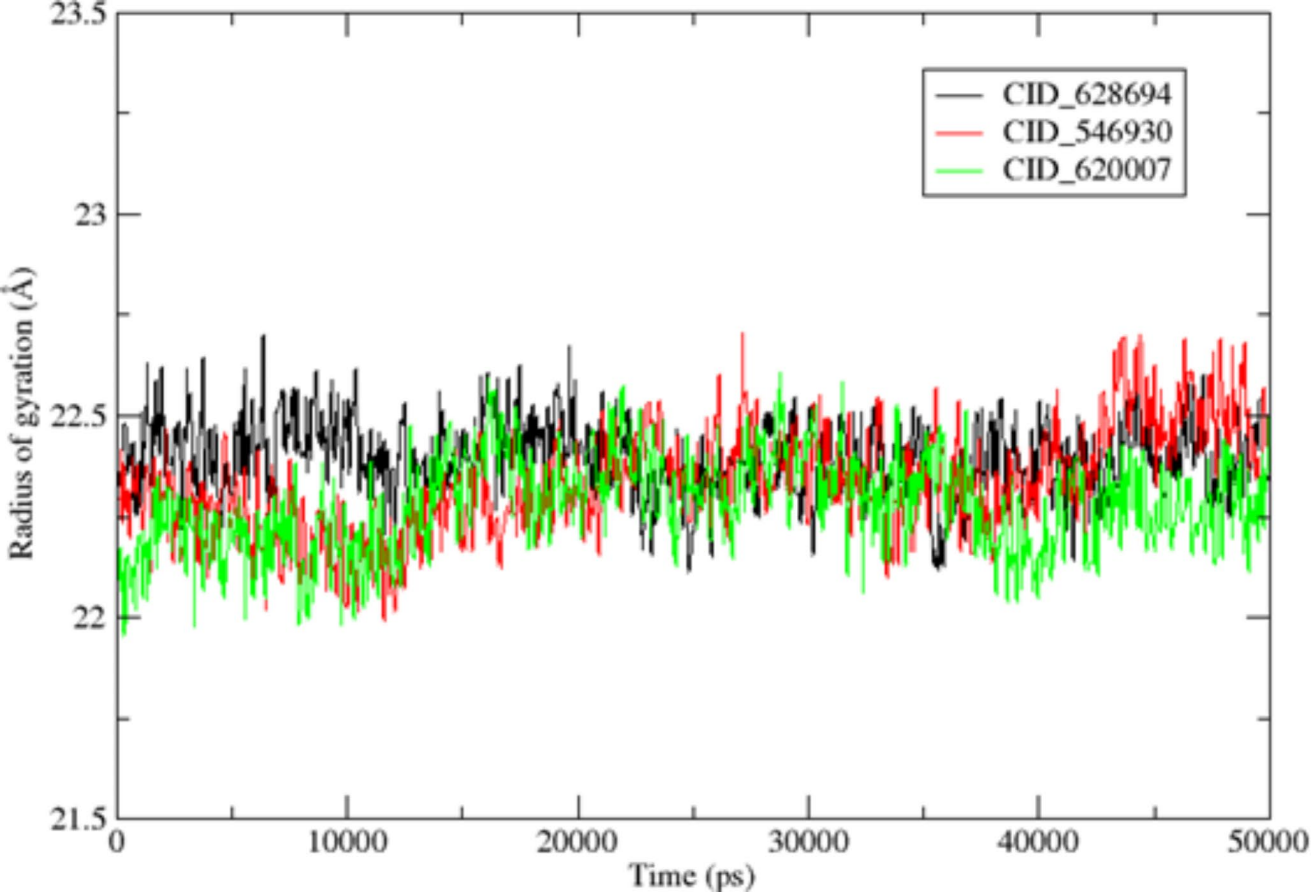
The Radius of gyration (Rg) values for PBP2a when bound to the chosen ligands—specifically, PBP2a─CID_628694, PBP2a─CID_546930, and PBP2a─CID_620007

The radius of gyration (Rg) measures the compactness of the protein–ligand complex, reflecting whether the protein maintains its structural integrity over time. For all three complexes, the Rg values remained relatively constant throughout the 50 ns simulation, indicating that the polypeptide backbone of PBP2a retained its folded structure in the presence of the ligands. The stable Rg values suggest that none of the ligands induced significant unfolding or destabilization of PBP2a, further corroborating the findings from the RMSD and RMSF analyses. This stability is crucial for potential inhibitors, as it implies that the ligands do not cause detrimental conformational changes that could compromise the functionality of PBP2a beyond inhibiting its active site (Fig. 7).

The combined results from the docking and MD simulation analyses provide a strong foundation for drug design. The stability of the protein–ligand complexes, particularly for CID_628694 and CID_546930, supports their potential as ‘lead’ compounds for further development. The MD simulations confirm the docking results, validating the interactions and energy values calculated in the static docking environment. This reinforces the value of CID_628694 as a highly potent inhibitor of PBP2a due to its stable binding and minimal deviation in both RMSD and RMSF. The stability observed in these MD simulations also suggests that these compounds are well-suited for in vitro testing. Compounds demonstrating stability in dynamic simulations will likely exhibit binding solid affinity and inhibitory activity in natural biological systems. Furthermore, the understanding gained from the MD simulations can inform the structure-based optimization of these ligands. By analyzing the vital interactions and stable regions within the protein–ligand complexes, modifications can be made to enhance binding affinity and pharmacokinetic properties, such as solubility and bioavailability.

## Conclusion

This study successfully identified the mecA gene from MRSA from clinical and environmental samples, reporting a prevalence rate of 35.5%. Molecular analyses confirmed the presence of the *mecA* gene, which underpins MRSA's resistance to β-lactam antibiotics. Phytochemical investigations of *Acacia nilotica* and *Mangifera indica* extracts identified bioactive compounds with antimicrobial potential. In vitro assays demonstrated inhibitory activity against MRSA, with minimum inhibitory concentrations (MIC) ranging from 0 to 6.20 mg/mL, although these concentrations may vary under different conditions. *In silico* molecular docking studies, we identified several phytochemicals with favourable binding affinities to the *mecA*-encoded protein PBP2a, with three compounds—CID_628694, CID_546930, and CID_620007—exhibiting strong interactions and stability within the protein's active site. Molecular dynamics simulations also validated the stability of these interactions, which supports our findings that these compounds could serve as potential PBP2a inhibitors. However, the docking and simulation results are predictive and require further experimental validation. While these findings indicate the potential of naturally derived phytochemicals as alternative therapeutic agents against antibiotic-resistant MRSA, the study has certain limitations. The efficacy of these compounds has only been demonstrated under in vitro and silico conditions. The phytochemical extraction methods and in vitro assay also need further validation. Future studies should focus on detailed in vitro and in vivo biological assays to confirm the therapeutic viability of these compounds and determine their pharmacokinetic and toxicological profiles.

## Data Availability

No datasets were generated or analysed during the current study.
